# Methods of multi-indication meta-analysis for health technology assessment: A simulation study

**DOI:** 10.1017/rsm.2025.10037

**Published:** 2025-10-01

**Authors:** David Glynn, Pedro Saramago, Janharpreet Singh, Sylwia Bujkiewicz, Sofia Dias, Steve Palmer, Marta Ferreira Oliveira Soares

**Affiliations:** 1 CÚRAM Research Ireland Centre for Medical Devices, University of Galway, Galway, Ireland; 2 Health Economics and Policy Analysis Centre, University of Galway, Galway, Ireland; 3 Centre for Health Economics, University of York, York, UK; 4 Biostatistics Research Group, Department of Population Health Sciences, University of Leicester, Leicester, UK; 5 Centre for Reviews and Dissemination, University of York, York, UK

**Keywords:** evidence synthesis, mixture models, multi-indication, simulation study, surrogacy

## Abstract

A growing number of oncology treatments, such as bevacizumab, are used across multiple indications. However, in health technology assessment (HTA), their clinical and cost-effectiveness are typically appraised within a single target indication. This approach excludes a broader evidence base across other indications. To address this, we explored multi-indication meta-analysis methods that share evidence across indications.

We conducted a simulation study to evaluate alternative multi-indication synthesis models. This included univariate (mixture and non-mixture) methods synthesizing overall survival (OS) data and bivariate surrogacy models jointly modeling treatment effects on progression-free survival (PFS) and OS, pooling surrogacy parameters across indications. Simulated datasets were generated using a multistate disease progression model under various scenarios, including different levels of heterogeneity within and between indications, outlier indications, and varying data on OS for the target indication. We evaluated the performance of the synthesis models applied to the simulated datasets in terms of their ability to predict OS in a target indication.

The results showed univariate multi-indication methods could reduce uncertainty without increasing bias, particularly when OS data were available in the target indication. Compared with univariate methods, mixture models did not significantly improve performance and are not recommended for HTA. In scenarios where OS data in the target indication is absent and there are also outlier indications, bivariate surrogacy models showed promise in correcting bias relative to univariate models, though further research under realistic conditions is needed.

Multi-indication methods are more complex than traditional approaches but can potentially reduce uncertainty in HTA decisions.

## Highlights

### What is already known?

- Oncology treatments, such as bevacizumab, are often used across multiple indications.
- HTA typically evaluate treatments within a single target indication, excluding broader evidence from other indications.
- Multi-indication meta-analysis methods have the potential to incorporate evidence across indications, potentially improving clinical and cost-effectiveness estimates.

### What is new?

- This study conducted a simulation to evaluate univariate and bivariate multi-indication synthesis models.
- Univariate methods can reduce uncertainty without increasing bias, especially when OS data are available for the target indication.
- Mixture models did not significantly improve performance and are not recommended for HTA.
- Under ideal conditions, bivariate surrogacy models showed promise in correcting bias when OS data are absent and there are outlier indications. Further research is needed.

### Potential impact for RSM readers

- The findings suggest that multi-indication synthesis methods can reduce uncertainty in HTA decisions.
- The study provides insights into when more complex synthesis methods might be beneficial, guiding future research and application in HTA.
- Readers can better understand the conditions under which different synthesis models perform well or poorly.

## 1 Background

Many oncology treatments are licensed for multiple indications. For example, bevacizumab is licensed in breast, colon, or cervical cancers, among other cancer types.^1^ However, licensing and health technology assessment (HTA) decisions for treatments are typically made on an indication-by-indication basis, relying only on evidence from the specific “target” indication of interest. For multi-indication treatments, incorporating evidence across indications, where appropriate, could strengthen clinical and cost-effectiveness estimates, reduce uncertainty in licensing and HTA decisions, and expedite patient access.^2^

A recent study^3^ examined the use of multi-indication evidence in HTA to estimate the effectiveness of bevacizumab on overall survival (OS) in advanced or metastatic cancers. The data consisted of 41 randomized controlled trials (RCTs) across seven cancer types, all trials reporting log hazard ratios (LHRs) for progression-free survival (PFS) and 36 also reporting LHRs for OS. The study applied univariate models synthesizing OS effects and bivariate surrogacy synthesis models, which established indication-specific surrogate relationships between the treatment effects on PFS and OS and synthesized surrogacy parameters. Models explored alternative sharing assumptions for the syntheses across indications. Independent parameters (IP), imposing no sharing of information; common parameters (CP), imposing full sharing of information; random parameters (RP), imposing partial sharing of information through an exchangeability assumption; or mixture models, also imposing partial sharing by allowing each indication to either be independent or to share information (using CP or RP), with the probability of sharing estimated from the data.

The data analyzed in Singh et al.^3^ showed small but consistent treatment effects on OS and PFS across studies, which did not seem to differ across indications.^3^ The application of univariate sharing models to this dataset, particularly under the CP assumption, significantly increased the precision of OS estimates compared to independent analysis. More complex mixture and bivariate models did not improve precision or fit and, in some cases, increased uncertainty. The limited performance of the more complex models was an unexpected finding but could potentially be explained by the limited between-indication heterogeneity in the dataset.

To support the broader application of multi-indication synthesis approaches a simulation study in which estimates can be compared against known true values of the estimand is needed to evaluate the performance of these methods. Further, it is necessary to assess the generalizability of the Singh et al. case study application to the variety of data structures encountered within HTA, including contexts of greater heterogeneity. More complex synthesis methods may perform better under larger levels of heterogeneity, in the presence of potential outliers and where evidence on the LHR for OS in the target indication is sparse, absent, or biased.^4^

The primary aim of this study was to assess the performance and impact of multi-indication synthesis methods compared to standard practices where evidence from other indications is excluded. Using simulation, we assessed these methods across various data structures considered relevant to HTA, focusing on their ability to predict treatment effects on OS for a specific target indication. Additionally, our work explored the conditions under which more complex synthesis methods, such as mixture and surrogacy models, might reduce bias and improve precision relative to simpler models.

To simulate the data, we used a previously developed data-generating model (DGM),^5^ based on a multistate model (MSM) of cancer progression with three states: pre-progression, progressed disease, and death. This MSM model, well established in oncology, has been used to represent natural history,^6^ ^–^ ^8^ quantify treatment effects,^9^ simulate surrogate relationships,^10^ and simulate trial data for sample size calculations.^5^ However, the LHR for PFS and OS analyzed by the synthesis models are not explicit parameters in the DGM but can be derived to obtain a joint distribution. Because the multi-indication synthesis models assume univariate or bivariate normality, the model specification differs between the DGM and the synthesis models. However, this is desirable as it results in realistic conditions for our simulation evaluation.

## 2 Multi-indication synthesis models

The following sections describe the multi-indication synthesis models considered in this paper; further details can be found in Singh et al.^3^

### 2.1 Univariate non-mixture models

In univariate models, the observed LHR for OS in study *i* and indication *j*, 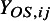 , are assumed to be normally distributed around their true value with standard errors *σ_OS,ij_*:

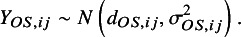



Random effects describe study results within each indication, meaning that the study-level effects were assumed normally distributed, with mean, *D_OS,j_*, representing the pooled effect for indication *j*. *τ_OS,j_* was the between-study, within-indication standard deviation:

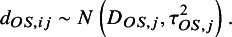



Assuming independent standard deviation parameters across indications (as in Ref. 3) is likely to require substantial data within each indication for precise estimation. Hence, we also explore the alternative assumption that this parameter is common across indications (*τ_OS,j_* = *τ_OS_*), assigning a weakly informative half-normal prior to the CP |*N* (0, 0.5^2^)|.^11^

To encode how information on *D_OS,j_* is related across indications (determining the sharing of information across indications), three different assumptions were explored (using either independent or common standard deviation parameters): IP model, imposing no sharing between indications; CP model, assuming equality across indications, that is *D_OS,j_* = *D_OS_*, in this way imposing maximal sharing of information; and random parameter (RP) model, imposing partial sharing by assuming *D_OS,j_* to be exchangeable across indications, *D_OS,j_* 
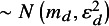
 . The RP model assumes full exchangeability, with the data determining the level of sharing across indications via the parameters of the common distribution. The between-indication standard deviation parameters quantify the level of heterogeneity between indications. Smaller standard deviation values suggest that the effect estimates are expected to be more similar across indications (with results of the RP model approximating those of the CP model), and larger values suggest that the effect estimates differ significantly from each other (with results of the RP model approximating those of the IP model).^3^

Six univariate models were therefore examined: IP*_τ_*, IP_τj_, CP_τ_, CP*_τj_*, RP*_τ_*, and RP*_τj_*. Table 1 summarizes how LHR OS in the target indication was predicted from each model. For all models (except CP), this depends on the availability of LHR OS data for the target indication. IP models rely on indication-specific evidence, and without this, predicted estimates of the LHR OS cannot be obtained. Because CP models use the common effect, in both cases, the LHR of OS can be predicted with or without LHR OS data in the target indication. In the RP model, shrunken indication-specific estimates were used to predict OS when OS data were present and the predictive distribution, 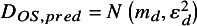 when OS data were absent.

**Table 1:** Synthesis models were investigated and the prediction of LHR on OS in the target indication from each model

Model summary focusing on between-indication sharing assumption		Prediction of LHR OS in target j*	
IP	Description	OS in target indication	No OS in target indication
IP	LHR OS: Independent	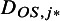	–
CP	LHR OS: Common	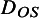	
RP	LHR OS: Exchangeable	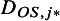	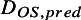
MCIP	LHR OS: Mixture of common and independent	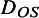	
MRIP	LHR OS: Mixture of exchangeable and independent	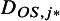	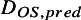
Bi-CP Unmatched	LHR PFS: independent	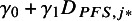	
	Surrogacy parameters: common
Bi-RP unmatched	LHR PFS: independent	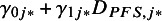	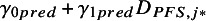
		Surrogacy parameters: exchangeable	
Bi-CP matched	LHR PFS: common	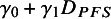	
		Surrogacy parameters: common
Bi-RP matched	LHR PFS: exchangeable	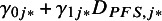	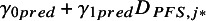
		Surrogacy parameters: exchangeable	

*Note*: Each listed model was run twice, assuming a common or independent between-study heterogeneity parameter. The prediction of the expected indication-specific effects from bivariate models does not use the conditional variance as this parameter captures variation across trials which is deemed unwarranted. j* = target indication; IP = independent parameters; CP = common parameters; RP = random parameters; MCIP = mixed common and independent parameters; MRIP = mixed random and independent parameters; Bi = bivariate; PFS = progression-free survival; OS = overall survival.

### 2.2 Univariate mixture models

Mixture models consider the effects in each indication to be either independent or from a shared distribution. The mixture probability reflected the probability that the effect in indication *j* came from the shared distribution, based on similarity with the other indication-level effects (i.e., a large probability would reflect strong similarity between effects). This was controlled by an indicator Bernoulli variable *c_j_*, which assumed the value of 1 for shared and 0 for independent. The Bernoulli probability parameter was estimated from the data. If the sharing component assumed CP across indications, this resulted in the mixture common and independent parameter (MCIP) model:

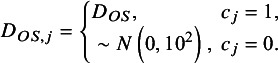



If the sharing component used RP across indications (that is exchangeable), this resulted in the mixture random and independent parameter (MRIP) model:

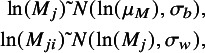



Mixture models allow the data to determine for each indication the plausibility of coming from a common distribution. For example, an indication with data that is extreme in relation to other indications is expected to estimate a small mixture probability value and, for this reason, is expected to make only a negligible contribution to the overall pooled parameter estimate.^3^

As with non-mixture models, the within-indication heterogeneity parameter was here also assumed either independent or common. This resulted in four univariate mixture model estimates: MCIP*_τ_*, MCIP*_τj_*, MRIP*_τ_*, and MRIP*_τj_*. The predicted effect in the target indication from each model was generated from the sharing component of the mixture model, the common CP for MCIP and RP for MRIP (see Table 1).

### 2.3 Bivariate (surrogacy) models

The bivariate models applied in Ref. 3 and further examined in this work extend Daniels and Hughes to consider multiple indications.^12^ In these models, a within-trial component describes the relationship between the treatment effects on a surrogate endpoint (PFS) and a final clinical outcome (OS) within an individual study, *i*:
(1)

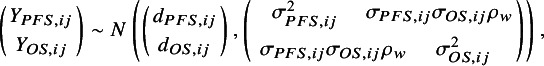

 where 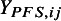 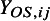 represent the observed LHR for PFS and OS, respectively, with standard errors 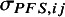 and 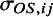 , and within study correlation, 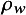 . Correlation was assumed common across studies and indications and assigned a weakly informative prior 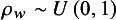 . A between-trial component describes the relationship between LHR OS and LHR PFS between studies (in the same indication, in this case), where a linear surrogate relationship is assumed between the true treatment effects on the surrogate endpoint 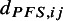 and the final outcome 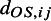 :
(2)

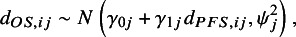

 where 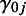 and 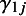 are the intercept and slope, respectively, in indication *j*. 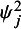 is the conditional variance of the relationship, which captures the extent to which a trial-specific LHR OS can be predicted from LHR PFS.

We examined sharing in all parameters in the surrogate relationship ( 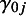 and 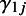 and 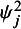 ). Two alternative sharing relationships were considered. A bivariate CP model (Bi-CP) assumed each surrogacy parameter as common across indications, and a bivariate RP model (BI-RP) assumed each as exchangeable, i.e. 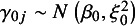 , 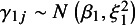 and 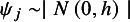 . Hyperparameters were assigned vague prior distributions.

Prediction of LHR OS for the target indication is summarized in Table 1 and requires estimates of the surrogate relationship from equation (2) and an estimate of LHR PFS for the target indication, 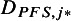 . The latter comes from the univariate models described in Sections 2.1 and 2.2 and can itself use different sharing assumptions. Following Singh et al., we considered “unmatched” and “matched” estimates. Matched estimates used the same sharing assumption (either CP or RP) for both the surrogate relationship and the univariate LHR PFS model. Unmatched estimates use a univariate IP model for LHR PFS data, and a sharing model for the surrogate parameters. For the LHR PFS estimate, we explored common or independent *τ* for the random effects within indications. This resulted in eight estimates from bivariate models being examined here, considering Bi-CP or Bi-RP, matched or unmatched, and with either independent or common within-indication heterogeneity parameter.

### 2.4 Simulation study methods

The simulation study was designed and reported using the “ADEMP” approach – aims, data generation, estimands, methods, and performance measures.^13^

#### 2.4.1 Aims

- Investigate if univariate (non-mixture) multi-indication sharing methods improve inferences over non-sharing methods by increasing precision, calibrating uncertainty, and maintaining low bias.
- Identify when different univariate sharing assumptions (common or exchangeable parameters) are appropriate and lead to significant precision gains, low bias, and well-calibrated descriptions of uncertainty.
- Explore when mixture models may reduce bias and calibrate uncertainty compared to non-mixture models.
- Explore when bivariate models may reduce bias and calibrate uncertainty over univariate models. This is exploratory and will be investigated under ideal conditions for surrogacy.

#### 2.4.2 Data generation

Generating multi-indication datasets requires the definition of the following elements: the DGM, the sets of parameter values examined, features of datasets (such as number of indications and study sample size), and the simulation process. We describe each of these elements in turn.

##### 2.4.2.1 Data-generating mechanism

**Figure 1:**
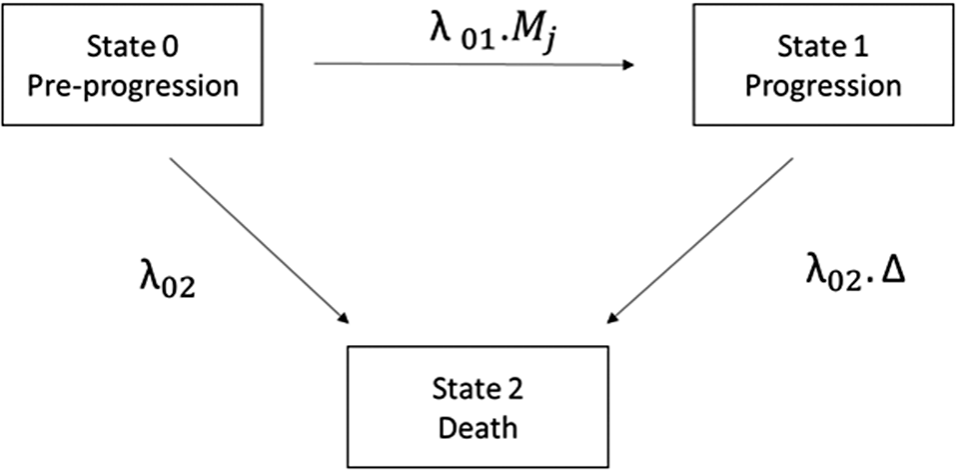
Data generating mechanism: three state MSM defining the relationship between PFS and OS in indication j. λ_01_ indicates the rate of progression, λ_02_ is the rate of pre-progression death, 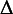 is the increase in mortality that results from progression. M_j_ is an indication specific multiplier which encodes how the new treatment impacts on the rate of progression. MSM = multi-state model; PFS = progression free survival; OS = overall survival.

The DGM uses the MSM from Erdmann et al. shown in Figure 1.^5^ This is a 3-state model that determines a structural relationship between progression and mortality. Patients start in the pre-progression state (state 0), where they are at risk of progression and death. The hazard of progression is *λ*_01_ *M_j_*, with λ_01_ representing the hazard under current practice and the multiplier, *M_j_*, representing how treatment impacts *λ*_01_ in indication j (slowing progression if *M_j_* < 1, accelerating it if *M_j_* > 1). The pre-progression hazard of death is *λ*_02_. The multiplier ∆ > 1 reflects the increase in mortality hazard after progression. Exponential hazards were assumed for all transitions. PFS is defined as time in state 0 and OS as the time spent in states 0 and 1. The MSM is used to (jointly) simulate time to progression and death for individual patients in a clinical study,^5^ which are subsequently used to define PFS and OS.

##### 2.4.2.2 Parameter value sets

The parameter value sets used in the MSM are summarized in Table 2; we here describe how those values were obtained. Estimates for MSM parameters unrelated to treatment, that is *λ*_01_ and ∆, were derived from data on the control arms of the largest RCTs for bevacizumab,^3^ and supported by an estimate of *λ*_02_ from Jansen et al.^9^ The control arms in most of these studies consisted of treatment with chemotherapy, representing the absence of targeted treatment. Between and within-indication heterogeneity on these parameters was not considered, so as to isolate the effects of treatment effect heterogeneity. Values were therefore derived independently by study and averaged to retrieve an overall estimate. Further details can be found in Supplementary Section A1.

**Table 2:** Design factors for the simulation study

	Description
MSM parameter mean values	*μ*_*λ*01_ = 0.097, *μ*_*λ*02_ = 0.01, *μ*_∆_ = 6.32, *μ_M_* = 0.6 on natural scale
Scenarios	Description
Variance parameter describing heterogeneity in the treatment effect, *M*	In all scenarios, between- and within-indication heterogeneity in *M* was varied, assuming coefficient of variation (CV) values of 0%, 7%, 15%, 30%, 50%. Other parameters (*μ*_*λ*01_, *μ*_*λ*02_, *μ*_∆_) were assumed independent by indication. These were not assumed heterogeneous within indications. Values were therefore kept constant across studies in the same indication.
Outlier indications	*No outlier indications*: All indications, including the target, were assumed exchangeable. *M_j_* values were randomly sampled from a common distribution based on a prespecified common mean and the between-indication heterogeneity parameter value. *One moderate (nontarget) outlier indication*: Same as above for all indications except the second largest indication, for which the *M* value was fixed at the 95% percentile (1.96*σ_b_*) of the between-indication heterogeneity distribution, rather than being randomly sampled. *One extreme (nontarget) outlier indication*: Same as above but the outlier indication had an *M* that was 6*σ_b_* away from the mean. *Outlier target indication*: Same as the no outlier indication scenario, but the *M* for the target indication was centered at the 95% percentile (1.96*σ_b_*) of the distribution describing between-indication heterogeneity in *M*.
Size of evidence base	*Small*: Four indications. Three indications reporting PFS and OS for 3, 2, and 1 study, respectively. 1 indication reporting PFS only. *Medium*: Six indications. Five indications reporting PFS and OS for 7, 3, 3, 2, and 1 study, respectively. One indication reporting PFS only. *Large*: Eight indications. Seven indications reporting PFS and OS for 9, 8, 6, 3, 2, 1, and 1 study, respectively. One indication reporting PFS only.
Target indication	*With OS*: Target indication had both OS and PFS data. *Without OS* [Results only presented in the appendix]: Target indication had only PFS data.

Treatment effects, M, were assumed to exhibit heterogeneity, both between- and within indications. The treatment effects were described using distributions, and simulated values were drawn from the nested log Normal distributions shown in the equations below:

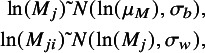

where *M_j_* is the treatment effect for indication *j*, sampled from a log normal distribution with mean *μ_M_* (the central treatment effect estimate across all indications) and standard deviation *σ_b_* representing between-indication heterogeneity. *M_ji_* is the treatment effect in study *i* (within indication *j*), sampled from a log normal distribution using the sampled *M_j_* as its mean value and a predefined value of *σ_w_* representing the within-indication standard deviation.

A value of 0.6 was used for *μ_M_* to represent a moderate treatment effect in oncology HTA; the heterogeneity parameters, *σ_b_* and *σ*_*w*,_ were defined using coefficients of variation (CV = *σ*/abs(*μ_M_*)) to ensure that conclusions are as independent as possible from the specific value of *μ_M_*. CV values examined were 0%, 7%, 15%, 30%, and 50%. A CV of 50% (the maximum considered) corresponds to a distribution where approximately 2.5% of simulated *M_j_* values exceed 1, indicating harm. Since this study focuses on approved indications under regulatory standards, higher probabilities of a treatment being harmful in a particular indication are unlikely.

The way treatment effect heterogeneity was defined means that treatment effects vary across indications but are centered around a common value, ln(*μ_M_*). Exchangeability can therefore be considered plausible. To test alternative assumptions, we ran scenario analyses introducing an outlier indication with a ‘divergent’ treatment effect value, not sampled from the same process. The treatment effect in the outlier indication was defined as being *μ_M_* = 1.96*σ_b_*, described as a ‘moderate’ outlier, with *M* drawn from the upper 95% interval for between indication heterogeneity. Additionally, an ‘extreme’ outlier was defined as *μ_M_* = 6*σ_b_*, with *M* drawn from the upper 99.99% interval. We explored the outlier being a nontarget indication, and to evaluate the advantages of bivariate methods, we also explored a scenario where the target indication is the outlier.

##### 2.4.2.3 Features of the multi-indication datasets

The number of indications and RCTs per indication in each scenario was based on the features of the bevacizumab dataset.^3^ Three cases were defined: a “large” evidence base with 8 indications representing the most developed dataset for bevacizumab, a “medium” base with 6 indications, and a “small” base with 4 indications. To reflect the HTA context, one indication was defined as the target and was assumed to include only one study which reported either PFS only or both PFS and OS.

The follow-up duration for all studies was set so that 80% of OS events occurred in the control arm. Following the studies in the Singh et al. case study, sample sizes were chosen so that each study had the power to detect a 0.7 hazard ratio with a 5% Type-1 error rate and 90% power (10% Type-2 error rate). The power calculation formula used was Lachin–Foulkes as implemented in the “gsDesign” package in R.^14^ We assumed an exponential rate of OS events and a two-arm balanced design, zero dropout, and instant accrual.

##### 2.4.2.4 Simulation process

As described above, study-level parameter values on *M* were sampled from their distributions. This generates a set of values with which to run the DGM simulation, generating simulated outcomes for individual patients (that is considering sampling uncertainty) according to a hypothetical trial with a set of defined features. The simulation used the ‘simIDM’ package in R,^15^ which implements the MSM model, generating individual values of time to progression and death. This was based on a nested set of competing risks experiments in a continuous time framework that implements the Erdmann et al. model.^5^ The simulated PFS and OS individual patient data from each study were analyzed by fitting Cox proportional hazard survival models to each outcome separately to estimate the LHRs for both outcomes. The Cox model was used because this is ubiquitous in practice. However, as shown by Erdmann et al.,^5^ LHR OS exhibits nonproportional hazards meaning it varies over time (except under very unrealistic assumptions). Therefore, the proportional hazards assumption can only hold approximately for OS.

#### 2.4.3 Estimand

An estimand is the specific quantity a study seeks to infer.^16^ ^,^ ^17^ In oncology HTA, a key estimand is the treatment’s relative effect on OS in the target indication that is the LHR OS. As noted above, LHR OS typically varies over time, so following Berry et al.,^18^ we defined the estimand as the expected LHR OS over study duration in the target indication. This was similar to restricted expectation and was based on the MSM parameters, *λ*_01_, *λ*_02_, ∆, and *M*. The true estimand value was calculated as a weighted average of the true LHRs at discrete time points (1.5 day intervals) weighted by the proportion of events expected within each interval.^18^ ^,^ ^19^

#### 2.4.4 Methods

We simulate 1000 multi-indication datasets as described in Section 3.2. Each of the statistical models described in Section 2 were fit to these datasets, using Markov Chain Monte Carlo (MCMC) in R JAGS (Just Another Gibbs Sampler). Three chains, a 50,000 burn-in, and 150,000 iterations were considered.^20^ Two criteria were used to assess MCMC convergence: Option 1 required 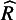 < 1.1 for all parameters,^21^ while Option 2 required 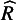 < 1.1 for only the model components used for prediction (see Supplementary Section A3). Runs that did not meet Option 2 were dropped. All analysis was carried out using the Viking cluster, a high-performance computer resource provided by the University of York. The simulation code is available at: https://github.com/david-glynn/Multi-indication-simulation. DOI:10.5281/zenodo.14849817.

#### 2.4.5 Performance measures

The performance of each multi-indication analyses model was evaluated using the following metrics (on the log hazard scale): bias (average error), coverage (the proportion of times that the true effect lies within the 95% credible interval which should be 0.95), and empirical standard error (SE) (variance of the estimate across iterations).^13^ To keep the paper concise, the latter metric is only shown in Supplementary Material. The strength of sharing for each method was assessed using the “splitting SE ratio,” which is the ratio of the SE estimate from a sharing model to that from the non-sharing model (IP_τ_).^22^ A value less than 1 indicates a gain of strength. These metrics were chosen to understand the statistical reliability of the methods and the magnitude of potential reductions in uncertainty. Monte Carlo errors were calculated for each metric.^13^

## 3 Results

We compared the performance of 18 synthesis models (each of the approaches in Table 1 evaluated twice, assuming common or independent random-effect heterogeneity parameters) across 600 scenarios, defined by factorially varying the design factors in Table 2. To keep the results section succinct, the figures shown in the main text are selected for relevance with the full results available in Supplementary Section A4.

### 3.1 Univariate (non-mixture) models

**Figure 2:**
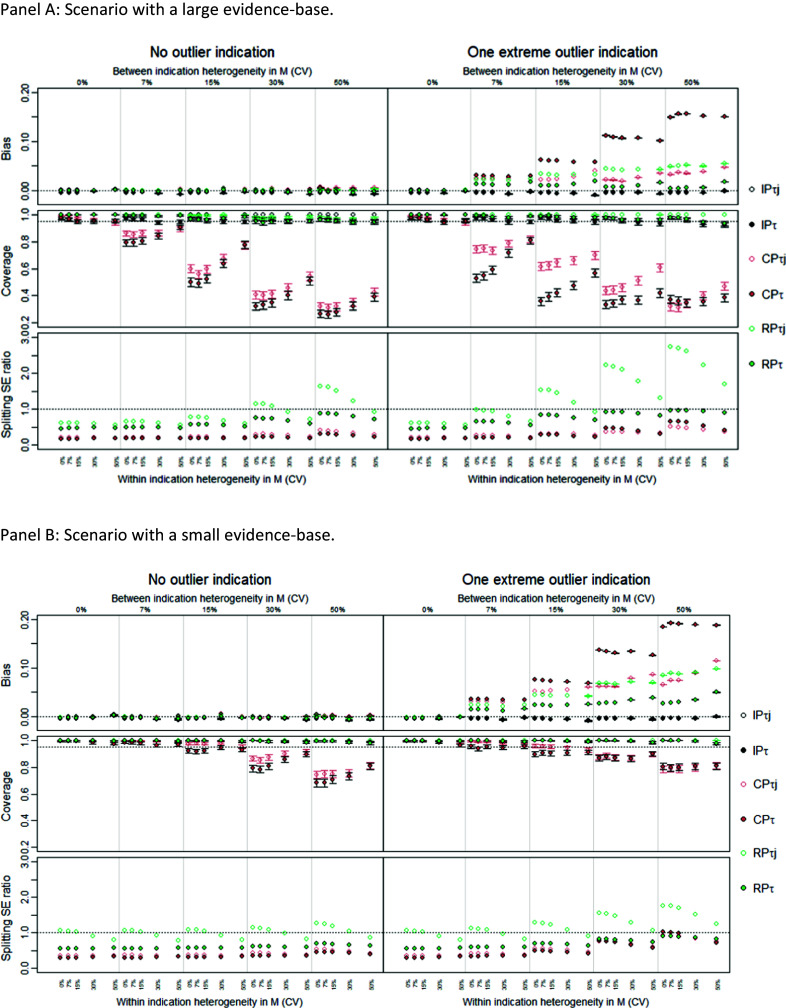
Performance measures comparing univariate non-mixture models with OS in the target indication. Results are shown for technologies with large (Panel A) and small (Panel B) evidence bases. The left panels show performance when there are no outlier indications, the right panels show performance for when the second largest indication is an outlier. Results are shown for all combinations of within indication heterogeneity (CV 0%, 7%, 15%, 30%, 50%) and between indications (CV 0%, 7%, 15%, 30%, 50%). CV = coefficient of variation; IP = independent parameters; CP = common parameters; RP = random parameters; τ = common within indication heterogeneity; τj = independent within indication heterogeneity.

In this section, we examine simulation results for the six univariate non-mixture models considered – the IP, CP, and RP models, assuming either *τ_j_* or *τ* – applied to the case where there is OS data in the target indication. The models showed a high convergence rate (>95%) across all analyses (for both convergence options 1 and 2). Figure 2 displays the performance metrics (*y*-axis) for the large and small evidence bases in panels a and b, respectively. The left side of each panel shows scenarios without outlier indications, while the right side shows scenarios with one extreme (nontarget) outlier indication. The *x*-axis is divided into five sections, corresponding to levels of between-indication heterogeneity in *M*, ranging from CV = 0% to 50% (top *x*-axis). Within each of these sections, scenarios vary by within-indication heterogeneity, from CV = 0% to 50% (bottom *x*-axis).

We first examine the case with no outlier indications (left plots in panels a and b, Figure 1). Results show that all univariate models examined were approximately unbiased (bias values close to zero), across all levels of within- and between-indication heterogeneity. This was expected as, in the absence of outliers, the DGM implies exchangeability in treatment effects across all indications, including the target. This means that, even under heterogeneity, the indication estimates are centered around a common value. This is well captured by the multi-indication models.

In the large evidence base (panel A), CP models were “well calibrated” for coverage at low levels of between-indication heterogeneity (CV ≤ 7%) that is the 95% interval included the true value ≈ 95% of the time. However, they were “overconfident” at higher heterogeneity levels that is they included the true value more than 95% of the time. RP models were anticipated to adjust for heterogeneity and remain well calibrated across the range of heterogeneity levels. However, they were underconfident at low heterogeneity and improved only at higher levels of heterogeneity. This may be due to identifiability issues for the within- and between-indication heterogeneity parameters, likely caused by the fact that data are sparse at the indication level leading to undue dominance of the “weakly informative” prior.

Sharing models showed lower splitting SE than non-sharing (IP) models in the large evidence base (panel A), meaning they borrowed more strength. CP models shared more strongly than RP models, with RP models showing only modest precision gains at higher levels of heterogeneity (CV ≥ 30%). In the small evidence base (panel B), the splitting SE increased for all models, and the overconfidence in coverage seen in CP models at high heterogeneity was less pronounced.

We now examine the case where there is an outlier (nontarget) indication (right plots in panels a and b, Figure 1). As expected, the IP model remained unbiased, but the sharing models showed bias. By virtue of the way the outlier was defined, the magnitude of bias depended on the level of between-indication heterogeneity. Among sharing models, RP*_τ_* showed the least bias because its shrunken estimate gives more weight to the indication-specific data, especially in cases of conflict. The effect of the assumption of common versus independent heterogeneity parameters on bias differed between RP and CP models: Common heterogeneity imposed higher bias with CP and lower bias in RP. The effect of such an assumption is complex but seems to increase the precision of smaller indications, and in CP, this seems to increase the influence of the outlier in overall estimates, while in RP, this seems to decrease the shrinkage of the target indication and, in this way, reduces bias.

The coverage and splitting SE results showed that, with an outlier, the strength of sharing of analysis models is reduced, especially with RP models, which inflate the between-indication heterogeneity to account for the divergence in the result of the outlier indication.

Overall, results for data structures without OS data in the target indication (Supplementary Section A4.1.2) were similar to those presented here, except for RP models. In the absence of OS data, RP models use the predictive distribution to generate an estimate for the indication, instead of the shrunken estimate used when there is OS data (Table 1). This leads to higher uncertainty and introduces potential for bias when there is an outlier among the nontarget indications.

### 3.2 Exploratory analyses of univariate mixture models under ideal conditions

According to convergence options 1 and 2, mixture models converged well (<5% non-convergence) except for the MCIP models when used to analyze small evidence bases (up to 32% non-convergence). The lack of convergence was associated with cases where the posterior sample space entered the region where *c_j_* = 0 for all *j*. In this region, the common effect cannot be estimated, and its value is therefore drawn from its prior. This generates discontinuity in the posterior chain and results in a large value for 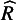 .

**Figure 3:**
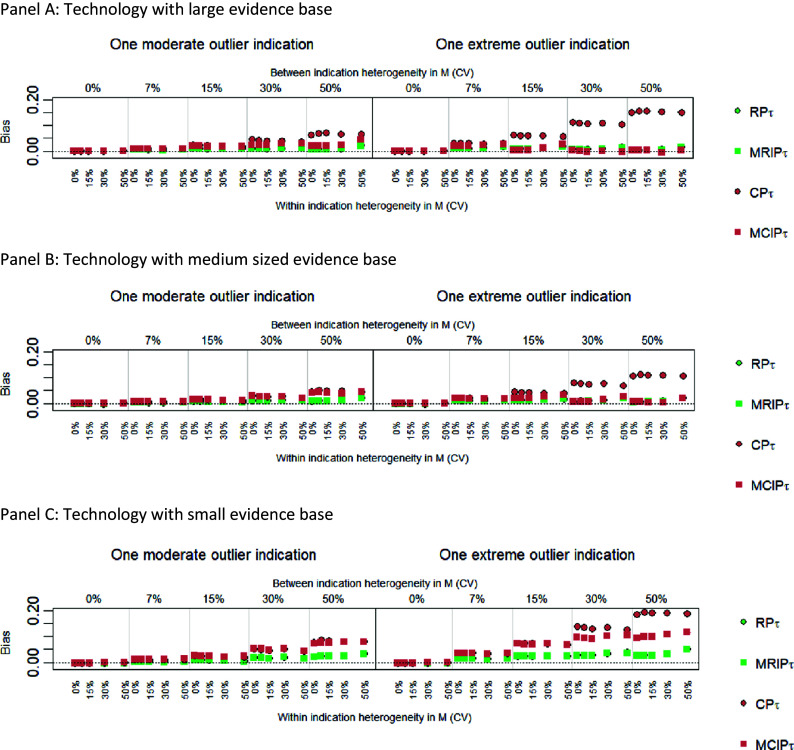
Comparing bias for all univariate mixture models and RP_τ_ with OS in the target indication. Results are shown for a large (Panel A), medium (Panel B) and small (Panel C) evidence base. The left panels show performance when the second largest indication is a moderate outlier, the right panels show results with an extreme outlier indication. Results are shown for all combinations of within indication heterogeneity (CV 0%, 7%, 15%, 30%, 50%) and between indications (CV 0%, 7%, 15%, 30%, 50%). CV = coefficient of variation; CP = common parameters; MCIP = mixed common and independent parameters; RP = random parameters; MRIP = mixed random and independent parameters; τ = common within indication heterogeneity.

To explore when the mixture models might overcome the limitations of non-mixture models, we restrict the presentation of results in the main paper to models that assume a common within-indication heterogeneity parameter, we include only datasets with an outlier indication, and we examine model performance only in terms of bias. Full results are, however, available in Supplementary Section A4.1. Figure 3 compares bias for MCIP and MRIP with their non-mixture counterparts, CP and RP, for datasets with a moderate (left side panel) and an extreme outlier (right side panel). Across all scenarios examined, mixture models presented no bias improvement over the RP model, although RP already presents relatively low levels of bias. MRIP presents similar results to its non-mixture counterpart, RP, across all scenarios. In contrast, the MCIP could only reduce bias in relation to CP when the outlier was extreme, when the evidence base was medium or large, and under high between-indication heterogeneity scenarios (≥30% CV). This pattern also held when there was no OS in the target indication.

### 3.3 Exploratory analysis of bivariate (surrogacy) models under ideal conditions

All surrogacy models converged well under the criteria in option 2. However, RP surrogacy models showed severe convergence issues under the criteria in option 1 (in more than 99% of runs), indicating difficulties with the identification of parameters not used for prediction, which may limit the reliability of these models.

For clarity, we only present results here for unmatched Bi-CP and Bi-RP compared to the univariate models IP and RP, all under the assumption of a common heterogeneity parameter, and applied to the case where there is OS in the target indication (full results are available in Supplementary Section A4.1). The matched bivariate models are presented in Supplementary Material only because, in cases where bias is small, the strength of sharing is weak (Bi-RP), or the coverage is inappropriate (Bi-CP). Further, the use of independent within-indication heterogeneity parameters was omitted as it produces highly uncertain LHR PFS predictions leading to highly uncertain OS predictions.

**Figure 4:**
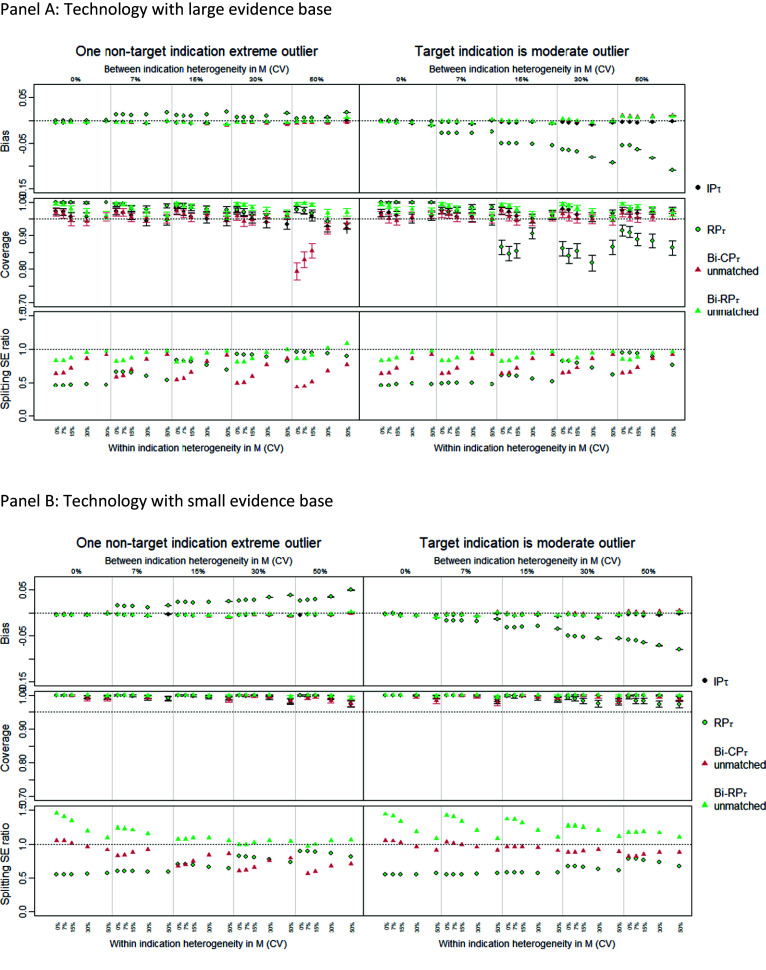
Performance measures comparing unmatched bivariate models (Bi-CP_τ_ and Bi-RP_τ_) and the univariate models IP_τ_ and RP_τ_ when there is OS in the target indication. Results are shown for large (Panel A) and small (Panel B) evidence base. The left panels show performance when the second largest indication was an outlier indication, the right panels show performance for when the target indication was an outlier indication and there is within and between indication heterogeneity in λ_01_, λ_02_ and 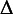 . Results are shown for all combinations of within indication heterogeneity (CV 0%, 7%, 15%, 30%, 50%) and between indications (CV 0%, 7%, 15%, 30%, 50%). CV = coefficient of variation; CP = common parameters; RP = random parameters; unmatched = use an independent parameters model to estimate progression free survival; τ = common within indication heterogeneity; τ_j_ = independent within indication heterogeneity.

Figure 4 presents results with a large and small evidence base and with a nontarget indication as outlier (extreme outlier) and with the target indication as outlier (moderate outlier). In the case where the nontarget indication is an outlier (left-side panel), surrogacy models generate OS predictions with low-level bias, suggesting that the IP estimate of LHR PFS is unbiased and that the surrogate relationship exists and is identified reliably. Bi-CP borrowed more strongly than Bi-RP. Critically, both models, however, showed little improvement in precision (splitting SE) compared to not sharing, IP, except for the Bi-CP model under high between-indication heterogeneity. This limits the potential value of the sharing on surrogate relationships.

Where the target indication is the outlier (right-side panel), RP becomes biased even when there is OS in the target indication, but both Bi-RP and Bi-CP were able to resolve such bias. When there is OS in the target indication, neither the bivariate model reduced uncertainty relative to IP except for the Bi-CP when applied to a large evidence base and under lower within-indication heterogeneity. This, again, limits the value of these models when the OS is available. In the absence of OS data on the target indication (see Supplementary Sections A4.1.2 and A4.2.2), IP estimates are not available, and bivariate models are the only option which provide an unbiased estimate for LHR OS.

## 4 Discussion

This article presents a simulation study exploring alternative multi-indication synthesis methods, considering a variety of data structures reflective of potential HTA contexts. This study focused on evaluating the use of these methods in supporting predictions of the effect of a hypothetical treatment on OS in a “target” indication by leveraging data from a broader evidence base across multiple indications. Although this study focuses on the impact of sharing information across indications, the results can be generalized to other sharing contexts, for example, sharing between drugs of the same class.^23^

We first explored simpler multi-indication synthesis methods that analyze LHR OS data directly (here termed univariate), including CP and RP models that respectively assume a common or exchangeable effect across indications. Our results showed that, when relative effectiveness across all indications was generated from the same process, with sharing being therefore plausible, these methods were unbiased and reduced uncertainty compared to using indication-specific evidence alone. This held even in the presence of heterogeneity between indications, although at higher levels of heterogeneity, the borrowing of information is reduced. Simulated scenarios where this heterogeneity was low showed that CP sharing worked well, leading to appropriate descriptions of uncertainty and strong borrowing of information. Contexts of low heterogeneity align with the assumption of a common effect underlying CP synthesis models. When this heterogeneity was high, RP was more suitable, providing better calibrated estimates and showing that the assumption of exchangeability underlying RP estimates can accommodate the increased heterogeneity. However, this required larger datasets as when small datasets were considered (six studies across three indications), RP was not well calibrated, generating higher uncertainty in predictions than was appropriate, that is underconfidence. In any case, high heterogeneity lends itself to limited borrowing of information and limited increase in the precision of estimates. The value of sharing in this context is constrained by this.

We examined the case where one of the nontarget indications was generated from a different, divergent process (an outlier indication) and should not be shared from. Multi-indication synthesis (also applied to the outlier) posed a risk of bias, except where OS data for the target indication were available and an RP model was used for analysis (assuming a common within-indication heterogeneity parameter). Without OS data in the target indication, all univariate methods were demonstrated to be biased. Our simulations assumed mature data (80% of events observed), but in HTA practice, OS data are often less mature, so real-world conclusions may fall between our “with OS” and “without OS” scenarios. Results for univariate non-mixture models indicate outlier indications can have important impacts on results. Multi-indication analyses, therefore, require that the plausibility of sharing between indications is assessed carefully, including consideration for heterogeneity within and between indications. There may be multiple sources of clinical and design heterogeneity, and careful consideration should be given as to whether these may generate statistical heterogeneity and impact on estimates of treatment effect. Taking heterogeneity in standard of care (SoC) across indications as an example: (1) If SoC is not an active treatment, then the assumption of a similar treatment effect across indications may be reasonable and not generate statistical heterogeneity. (2) If SoC is an active treatment, and the treatment of interest is an “add on”, then the assumption of an exchangeable additive treatment effect may be appropriate (this was the case for bevacizumab in the Singh et al.^3^ case study). (3) If the treatment of interest replaces an active SoC and if the effectiveness of SoC varies by indication, then this could generate effect heterogeneity. In this case, it may be possible to extend the multi-indication synthesis to a network meta-analysis (NMA) to address this. Further extension such as population and dose adjustment may be required in some cases to improve the plausibility of the exchangeability assumption.^24^ ^,^ ^25^ A further risk to the exchangeability assumption proposed for multi-indication analyses arises if manufacturers first launch treatments in indications in which they expect them to have the largest effects. This would result in a systematic ordering of treatment effects over time. Further research is required to establish the presence and magnitude of this effect.

After looking at simpler univariate models, we examined whether mixture models could identify and correct for the outlier indication. Mixture models are argued to be more robust to outliers.^11^ ^,^ ^26^ ^,^ ^27^ However, we found that, under the data structures typical in HTA contexts, these models were not able to identify the outlier and eliminate bias. Mixture models add complexity and are unlikely to provide improvements in practice, so they should not be used.

We also explored the performance of bivariate (surrogacy) multi-indication synthesis models in predicting LHR OS effects for a target indication under ideal conditions for surrogacy. When there is an OS in the target indication, both univariate and unmatched bivariate models can correct for bias due to outlier indications. Due to the additional complexity of surrogacy models and the limited added value, they may not be useful in this case. However, the potential gains from surrogacy are clearer when (i) the target indication itself is an outlier and (ii) there are nontarget outlier indications and no OS in the target indication (but there is PFS). In these cases, unmatched surrogacy models can provide low-bias estimates for HTA decision-making. This use of surrogacy has been considered elsewhere.^28^ ^,^ ^29^

Our simulation study used a mechanistic model (a three-state cancer progression MSM) to generate datasets, but analyzed these assuming univariate or bivariate normality of the LHR for PFS and OS. The mechanistic element provides a novel way to examine the accuracy of the commonly applied synthesis models, emphasizing that these may be misspecified. It is when analyzing the relationship between LHR PFS and OS that we believe misspecification may have a more marked influence from neglecting nonlinearity. Further research should explore the reliable conditions for linear surrogate relationships and the impact of deviations from linearity on the accuracy of the bivariate models used for analyses here, which are based on a linear surrogacy assumption. In examining the conditions for linear surrogacy, a critical consideration is the inclusion of heterogeneity in time to progression or pre-/post-progression survival, which was not considered in our study but is realistic, as differences in prognosis are expected between indications (such as between prostate and breast cancer) (see Supplementary Section A2). An alternative approach to imposing a linear surrogate relationship is to fit an MSM model directly; however, this is complex and uncommon in practice.^9^

Our work synthesizes HRs as metrics of relative treatment effect. HR was chosen because it is ubiquitous in HTA. It is often assumed to be constant with time (proportional hazards assumption) and therefore allows for the simpler case where the treatment effect is defined by a single parameter. In principle, the methods described in the article could be applied to effects both on the relative or absolute scales, but justifying the exchangeability assumption across indications may be challenging in the context of absolute effects. The DGM utilized in the study implies a mild violation of the proportional hazards (PH) assumption, but the methods tested were found to be robust to this violation. However, we did not consider more significant violations of PH such as those that can occur in the context of cancer immunotherapy treatments.^30^ More complex approaches such as fractional polynomials or piecewise exponential models may have utility in this context, and further research could consider extending these models to the multi-indication case.^9^ ^,^ ^30^

For multi-indication meta-analysis methods to be impactful in HTA, typical data structures must be considered. Multi-indication methods can provide the most support for decision-making when used early in the indication rollout when the number of approved indications are small. In HTA, there are often relatively few indications, few studies per indication, and immature evidence, especially for OS. This, combined with heterogeneity within and between indications, can account for the poor performance of highly parameterized synthesis models such as mixture models.

As with any simulation study, there are a number of ways to further extend and improve the generalizability of our findings, including extending the DGM to include heterogeneity in parameters other than the treatment effect, extending the scenarios evaluated or considering alternative estimands more relevant for HTA decision making that consider, for example, the joint estimation of PFS and OS or immaturity in data.

The univariate (non-mixture) sharing methods described here are a relatively simple extension to standard meta-analysis methods and can provide insight into the implications of different assumptions about sharing across indications. In some cases, ignoring the evidence in related indications will not be reasonable and result in more uncertainty in estimates than is warranted. The tools described here allow for assumptions to be explicit. This contrasts with the informal approaches often used in HTA which, under sparse evidence of impact on OS, rely on implicit assumptions with limited support. However, the application and interpretation of any statistical model requires a judgement on the plausibility of such models. In multi-indication HTA, because of the likely heterogeneity and the relatively small quantity of data (number of studies within indications), it is necessary that analyses are guided by clinical judgement on the plausibility of sharing from each indication. This could encompass selecting indications to include in the dataset or applying quantitative weights to indications based on their relevance to the target indication. Further research in expert elicitation is required on (1) how to gather and integrate these judgements^31^ and on (2) what information is required to support these judgments. On the latter, relevant information may include biological factors such as tumor genomic profile and treatment effect mechanisms.^32^ It will also be important to consider the source of heterogeneity between indications and whether this may be due to differences in clinical practice and/or trial protocols e.g., due to differences in the SoC or second-line treatment options.

## 5 Conclusion

This analysis has implications for HTA decision-making, which currently relies on single indication data to make decisions about multi-indication drugs. Our results showed that, for typical HTA contexts, univariate multi-indication methods (simple extensions of standard meta-analytic methods) can reduce uncertainty without inflating bias, particularly where there are OS data on the target indication. Mixture models add complexity and are unlikely to provide improvements in practice, so they should not be used. In cases where univariate models may become biased, such as with outlier target indications, bivariate surrogacy models show potential, but further research is necessary to understand their performance under more realistic conditions.

## Supporting information

Glynn et al. supplementary material Glynn et al. supplementary material

## Data Availability

This is a simulation study; all data can be generated using the code available at: https://github.com/david-glynn/Multi-indication-simulation. DOI: 10.5281/zenodo.14849817.

## Supplementary material

To view supplementary material for this article, please visit http://doi.org/10.1017/rsm.2025.10037.
