# Supplementary material for: Methods of multi-indication meta-analysis for health technology assessment: A simulation study
Source: Res Synth Methods. 2025 Oct 1;17(1):93–110. doi: 10.1017/rsm.2025.10037 (PMC12823204; doi:10.1017/rsm.2025.10037)
Supplement: Glynn et al. supplementary material [file S1759287925100379sup001.docx]

# A1. Estimating multi-state parameters based on median PFS and OS

In this section we describe our approach to find reasonable multi state model parameters to ground our simulation study. We do this using the bevacizumab case study described in Singh et al (2023). Specifically, we want to find values for $\lambda_{01}, \lambda_{02}$ and Δ which represent their mean value across the indications in the bevacizumab case study. As described in the paper mean value of M was assumed to 0.6 (μ_M_ = -0.511 on log scale) and a wide range of values of within and between heterogeneity were explored.

A “initial-progressed-dead” multistate model equivalent to the one used in the main paper is illustrated below. This differs from the main paper in that it does not include a treatment effect on progression (M) and the rate of post progression death is parameterised using $\lambda_{12}$ rather than as $\lambda_{12}$.Δ.


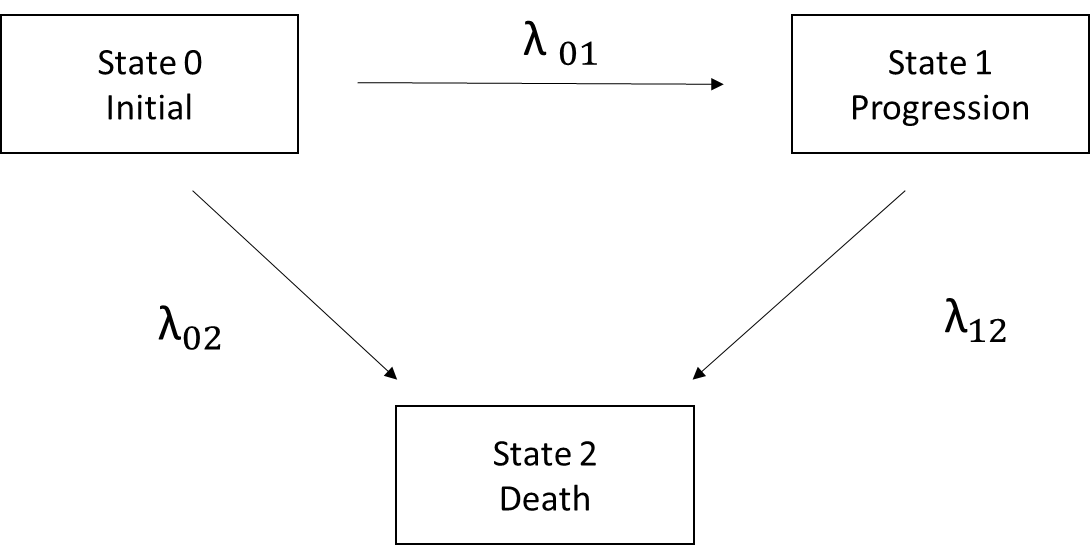


*Figure A1.1: “Initial-progressed-dead” multistate model as described in Erdmann et al (2025).*

With the objective of estimating transition rates for the model shown in Figure A1.1, median OS and PFS were extracted from the control arms of the larger studies within each cancer type (among those studies that reported both PFS and OS), see Table A1.1.

| **Cancer type** | **Publication** | **Line of treatment** | **Median, in months** | | $\boldsymbol{\lambda}_{\boldsymbol{01}}^{\boldsymbol{ctrl}}$ | $\lambda_{02}^{ctrl}$ | $\boldsymbol{\lambda}_{\boldsymbol{12}}^{\boldsymbol{ctrl}}$ | **Δ** |
| --- | --- | --- | --- | --- | --- | --- | --- | --- |
|  |  |  | **PFS ctrl** | **OS ctrl** |  |  |  |  |
| BRE | Brufsky (2011) | Second | 5.1 | 16.4 | 0.126 | 0.0102 | 0.072 | 7.0588 |
| BRE | Cameron (2008) | First | 5.8 | 24.8 | 0.109 | 0.0102 | 0.039 | 3.8235 |
| BRE | Miles (2013) | First | 8 | 31.9 | 0.076 | 0.0102 | 0.03 | 2.9412 |
| CER | Tewari (2017) | First | 6 | 13.3 | 0.105 | 0.0102 | 0.124 | 12.1569 |
| COL | Saltz (2008) | First | 8 | 19.9 | 0.076 | 0.0102 | 0.07 | 6.8627 |
| COL | Schmoll (2012) | First | 9.9 | 22.8 | 0.06 | 0.0102 | 0.064 | 6.2745 |
| COL | Bennouna (2013) | Second | 4.1 | 9.8 | 0.159 | 0.0102 | 0.161 | 15.7843 |
| GLIO | Chinot (2014) | First | 6.2 | 16.7 | 0.102 | 0.0102 | 0.08 | 7.8431 |
| GLIO | Wick (2017) | Second | 1.5 | 8.6 | 0.452 | 0.0102 | 0.107 | 10.4902 |
| GLIO | Gilbert (2014) | First | 7.3 | 15.7 | 0.085 | 0.0102 | 0.106 | 10.3922 |
| NSCLC | Sandler (2005) | First | 4.5 | 10.3 | 0.144 | 0.0102 | 0.16 | 15.6863 |
| NSCLC | Reck (2019) | First | 6.9 | 21.4 | 0.09 | 0.0102 | 0.054 | 5.2941 |
| NSCLC | Zhou (2015) | First | 6.5 | 17.7 | 0.096 | 0.0102 | 0.075 | 7.3529 |
| OFTPP | Oza (2015) | First | 17.5 | 58.6 | 0.029 | 0.0102 | 0.013 | 1.2745 |
| OFTPP | Aghajanian (2012) | Second | 12.4 | 35.2 | 0.046 | 0.0102 | 0.031 | 3.0392 |
| OFTPP | Burger (2011) | First | 10.3 | 39.3 | 0.057 | 0.0102 | 0.023 | 2.2549 |
| REN | Escudier (2007) | First | 5.4 | 19.8 | 0.118 | 0.0102 | 0.054 | 5.2941 |

*Table A1.1: This table shows the median PFS and OS values extracted from control arms of larger studies in the bevacizumab case study reported in Singh et al (2023). It also shows the multi state model parameters assumed or derived from these studies. OS = overall survival; PFS = progression free survival; BRE = breast; CER = cervical; COL = colorectal; GLIO = glioblastoma; NSCLC = non-small cell lung cancer; OFTPP = Ovarian, fallopian tube and primary peritoneal; REN = renal.*

To derive the multi-state parameters from the extracted median PFS results, we assume that PFS is a random variable which follows an exponential distribution. Therefore, median(PFS)=$\frac{log(2)}{\lambda}$, where λ is the rate parameter. In the case of the control arm of the multistate model λ = $\lambda_{01}^{ctrl}+\lambda_{02}^{ctrl}$. This results in the following formula:

$\lambda_{01}^{ctrl} =$ln(2)/median(PFS) - $\lambda_{02}^{ctrl}$.

From first-line treatment of adult patients with NSCLC in Jansen et al (2023) $\lambda_{02}^{ctrl}$= 0.0102. This was assumed constant across all studies. By plugging in $\lambda_{02}^{ctrl}$and the observed control arm median(PFS) from Table A1.1 above, we estimated $\lambda_{01}^{ctrl}$ for each study.

Given exponential transitions, Erdmann et al (Erdmann, Beyersmann et al. 2025) provide equations which deterministically relate the multi-state model parameters in Figure A1.1 ($\lambda_{01}, \lambda_{02}, \lambda_{12}$) to survival functions (and therefore medians, S(t) = 0.5) for PFS and OS. For each trial we used these equations and the values for $\lambda_{01}^{ctrl}$, $\lambda_{02}^{ctrl}$to back solve for the value of $\lambda_{12}^{ctrl}$ which is consistent with the median(OS) observed in the control arm of the trial. This was done using single parameter optimization in R.

The multi-state model described in the main text is defined in terms of $\lambda_{01}, \lambda_{02},$ Δ and M. Differences between the arms are defined using M so $\lambda_{01}=\lambda_{01}^{trt}$ . Pre-progression mortality was assumed constant across treatment arms so $\lambda_{02} = \lambda_{02}^{ctrl}$. Δ was defined as a multiplier for the change in mortality post progression, it was assumed common across arms so is defined as $\lambda_{12}^{ctrl}$/$\lambda_{02}$.

Because $\lambda_{02}$ was assumed to be constant at 0.0102, the mean value across indications is μ_λ02_ = 0.0102. To calculate the mean value of $\lambda_{01}$ and Δ across indications we need to first convert the parameters of interest in each study to the log scale. Then for each parameter we calculate the mean within each indication. Then, weighting each indication equally, we take the average of the indication means. This results in μ_λ01_ = 0.097 and μ_∆_ = 6.32 on the natural scale.

# A2. Linear surrogacy between log hazard ratios for progression free survival and overall survival

As shown in Erdmann et al (Erdmann, Beyersmann et al. 2025), under the conditions of the MSM described in the main paper, LHR OS and LHR PFS are a function of the MSM parameters and duration of follow-up (t). The LHR for PFS is given by:

LHR PFS$=ln\left( \frac{\lambda_{01}.M + \lambda_{02}}{\lambda_{01} + \lambda_{02}} \right)$.

The OS hazard (h_OS_) in the control and treatment groups are below:

$h_{OS, ctrl}= \frac{\Delta(\lambda_{01} + \lambda_{02}) - \lambda_{01}.\lambda_{02} + \Delta.exp((\lambda_{01} - \Delta).t)}{\Delta- \lambda_{01}.exp((\lambda_{01} - \Delta).t)}$,

$h_{OS, trt}=\frac{\Delta(\lambda_{01}.M + \lambda_{02}) - \lambda_{01}.M.\lambda_{02} + \Delta.exp((\lambda_{01}.M - \Delta).t)}{\Delta- \lambda_{01}.M.exp((\lambda_{01}.M - \Delta).t)}$.

Therefore, the LHR OS is the log of the ratio of these hazards.

LHR OS $= ln\left( \frac{h_{OS, trt}}{h_{OS, ctrl}} \right)$.

To understand when approximately linear surrogacy is possible, we simulated from the equations above. We begin with the mean parameters used in the case study (and based on the bevacizumab dataset): $\lambda_{01}$ = 0.097, $\lambda_{02}$ = 0.01, Δ = 6.32, M = 0.6 on natural scale. For each MSM parameter we explore a wide range of values, investigating the impact on LHR PFS, LHR OS and their joint distribution (i.e. the surrogacy relationship). Note that in using the above equations we are simulating the true underlying study level values with no sampling uncertainty.


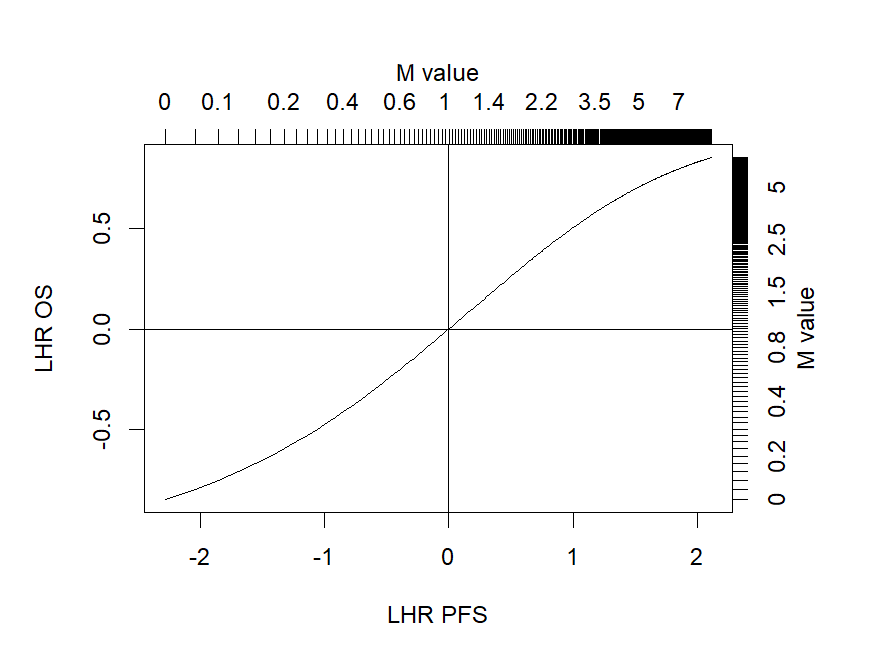

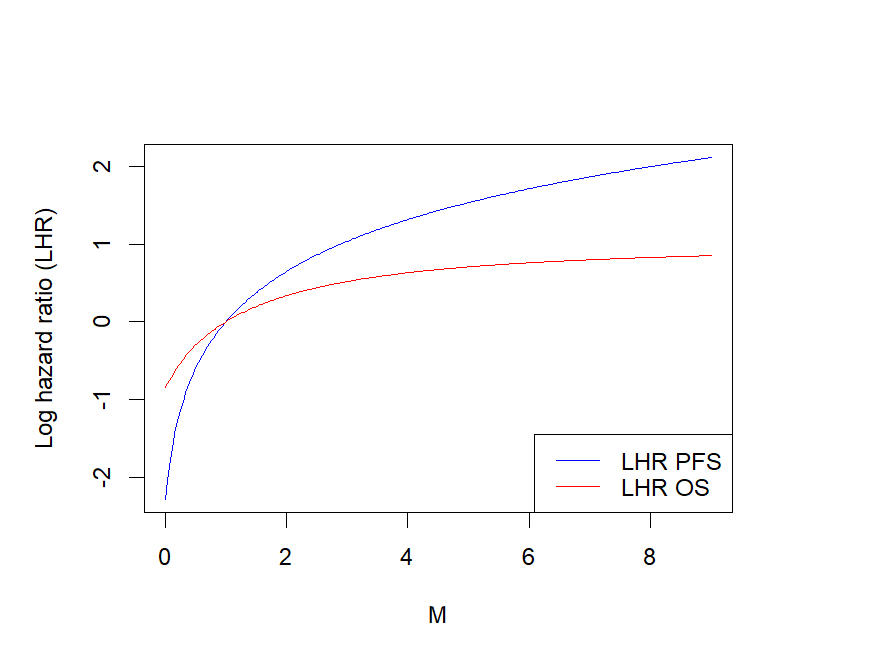


Above M is varied from 0.01 to 9 (natural scale). Surrogacy is approximately linear when the treatments have a similar effect i.e. M close to 1.


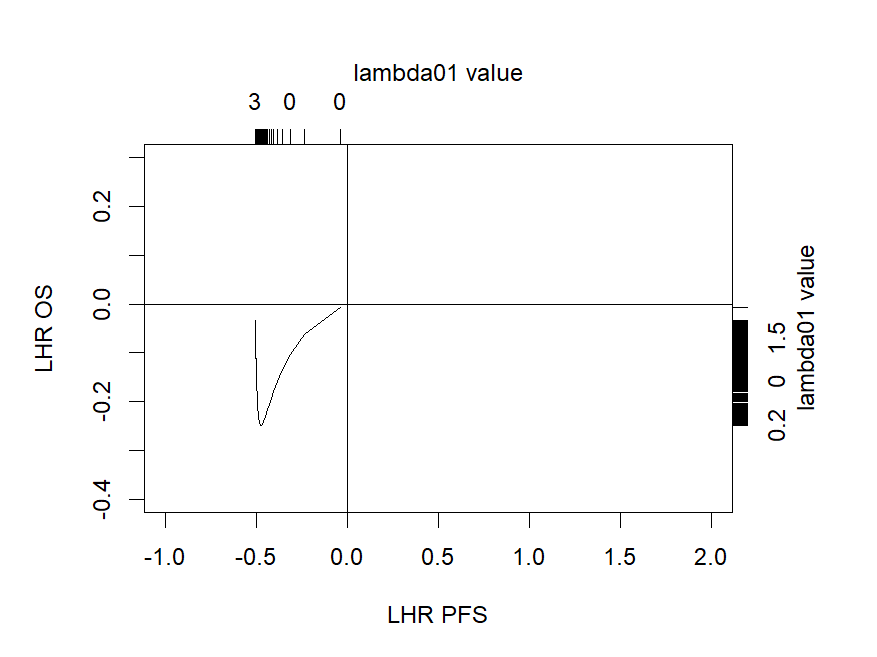

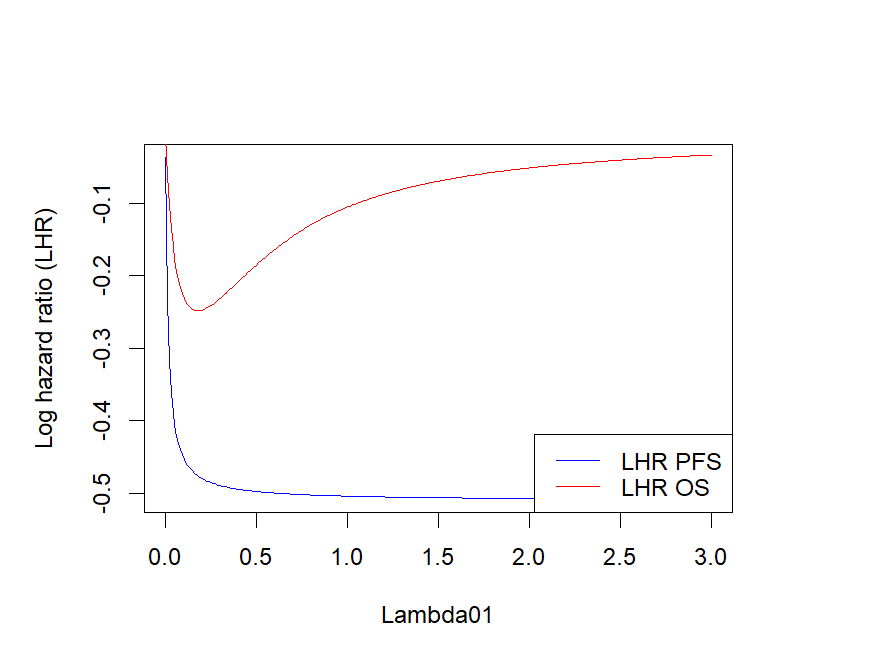


Above λ_01_ is varied from 0.001 to 3 (natural scale). The surrogacy relationship is non-monotonic. This is because LHR OS is non-monotonic in λ_01_ (see right hand graph).


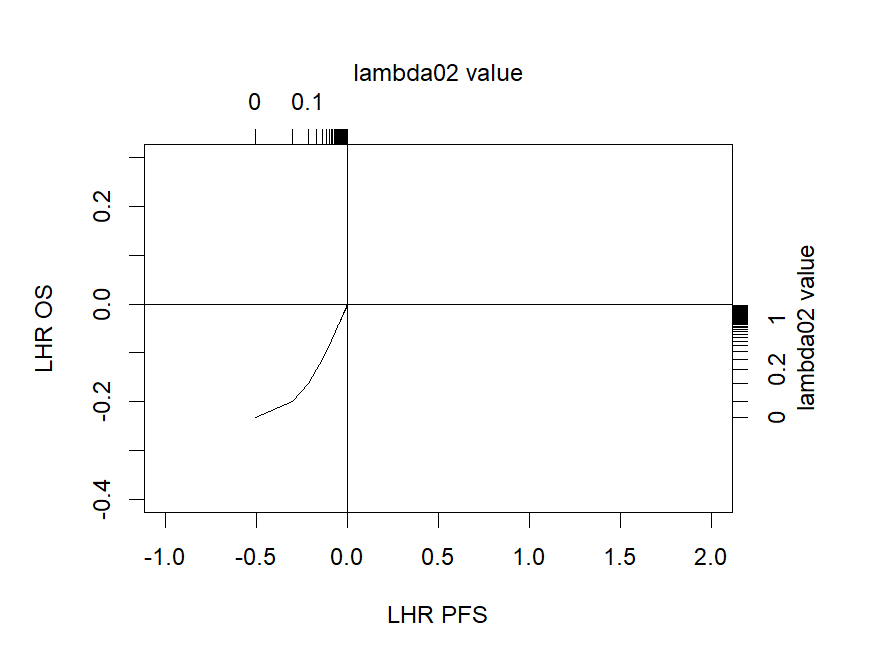

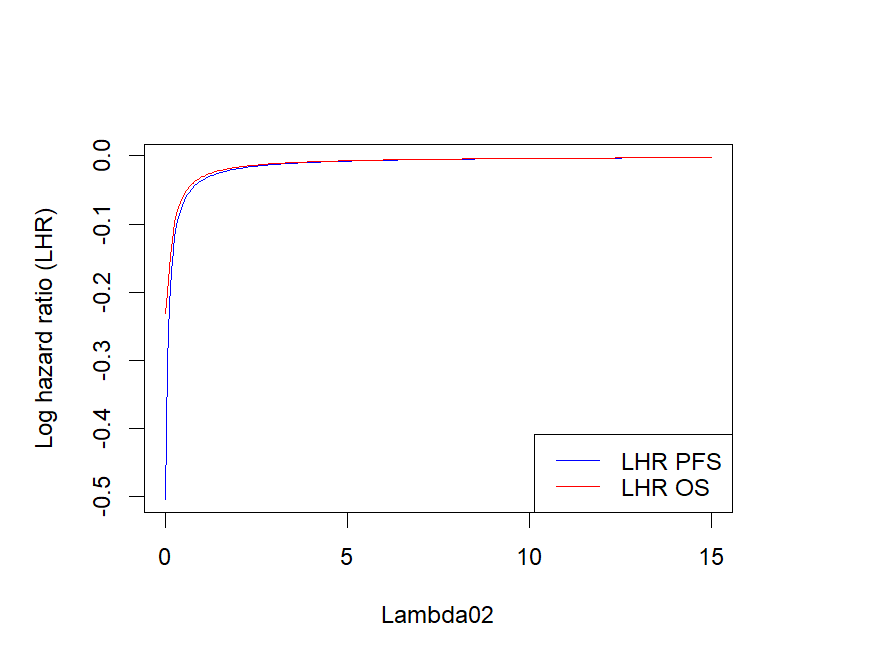


Above λ_02_ ranges from 0.001 to 15 (natural scale). There is an approximately linear component between LHR PFS = 0 and -0.25.


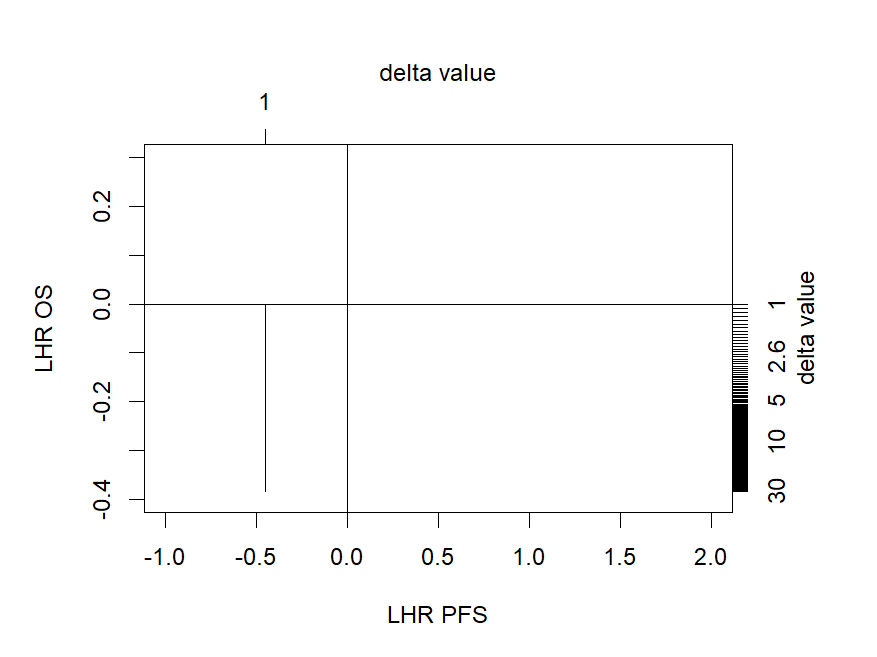

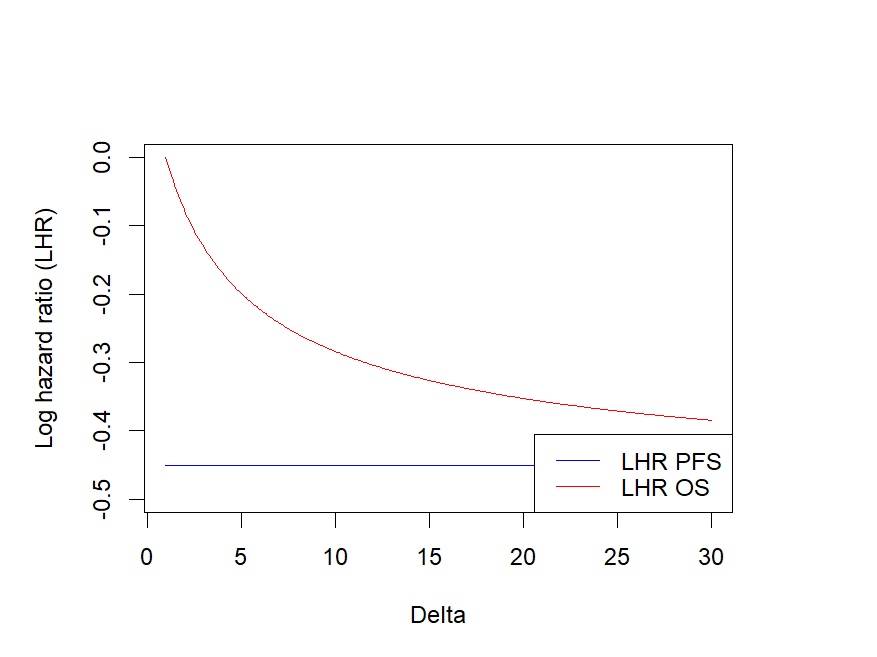


Above ∆ ranges from 1 to 30 (natural scale). Because this is the increase in the rate of mortality post progression it has no impact on PFS. This mean that there is no surrogacy relationship induced by variation in ∆.

This analysis indicates that variation in λ_01_, λ_02_ and M can be associated with approximately linear surrogacy regions. In each case this region does not extend over all values of the MSM parameter. This indicates that linear surrogacy cannot be assumed generally.

Note that because the MSM is non-linear, the results above depend on the mean values of all the parameters in the model so any generalisation is challenging.

# A3. Definitions of model convergence

**Option 1:** $\hat{\boldsymbol{R}}$ **< 1.1 for all parameters**

Fit model using JAGS then calculate $\hat{R}$ statistic for all model parameters. The model has converged if $\hat{R}$ < 1.1 for all parameters. Note that for mixture models “c” and “if branch” are included in the MCMC output but are indicators and not model parameters so $\hat{R}$ is not calculated for these model components.

**Option 2:** $\hat{\boldsymbol{R}}$ **< 1.1 for parameters used in prediction**

The principle behind this approach is that convergence only matters in those parameters which are ultimately used to make the prediction for the estimand of interest. For example, the mixture models include some parameters which will not be updated if the model considers them implausible, so it does not make sense to check the convergence of these parameters. To implement this option, we need to define the parameters used in prediction for each model, this information is included in Table 1 of the main paper.

# A4. All simulation study results

# A4.1 Outlier indications

## A4.1.1 With overall survival in target indication

### Univariate non-mixture

**Large dataset**


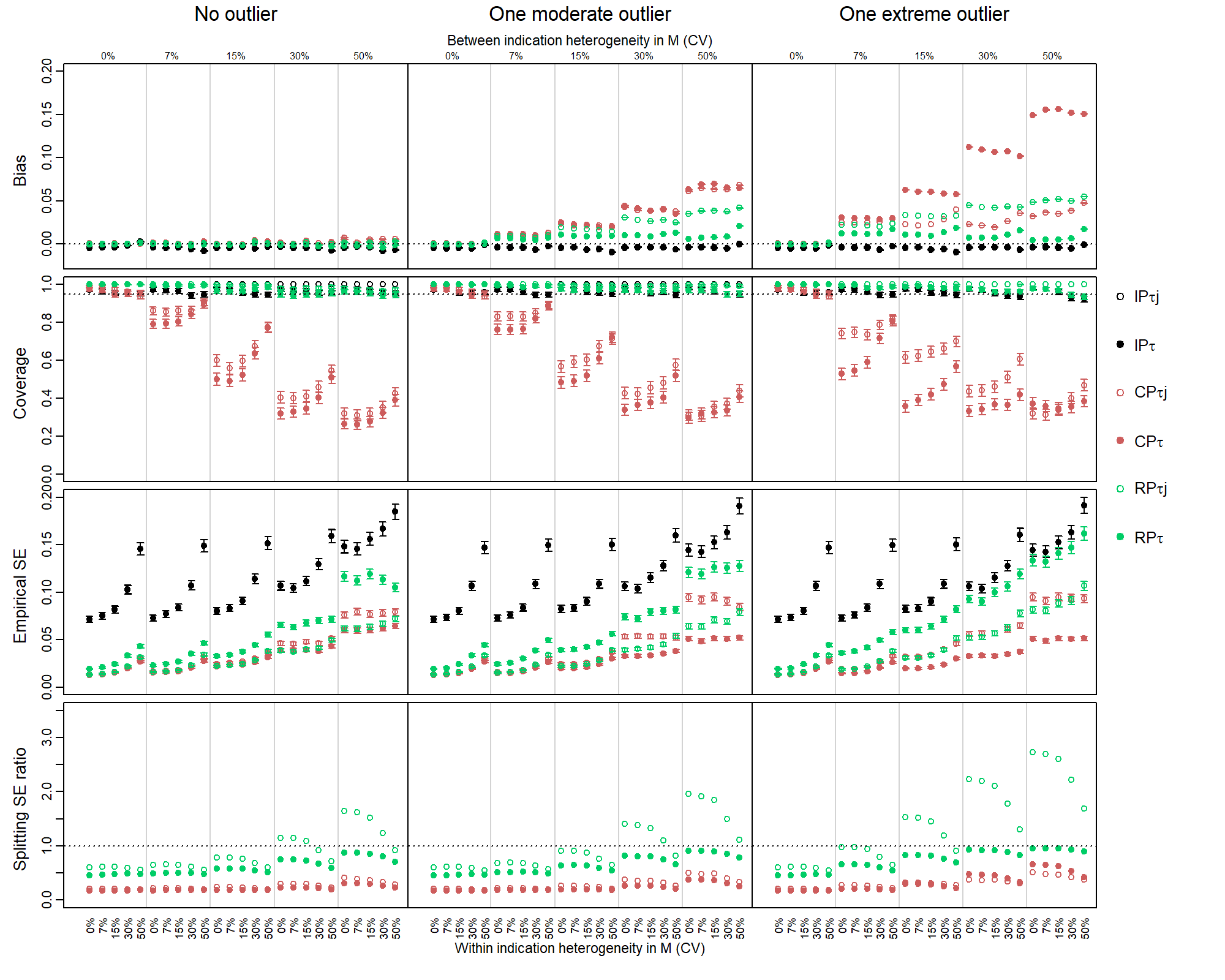


**Medium dataset**


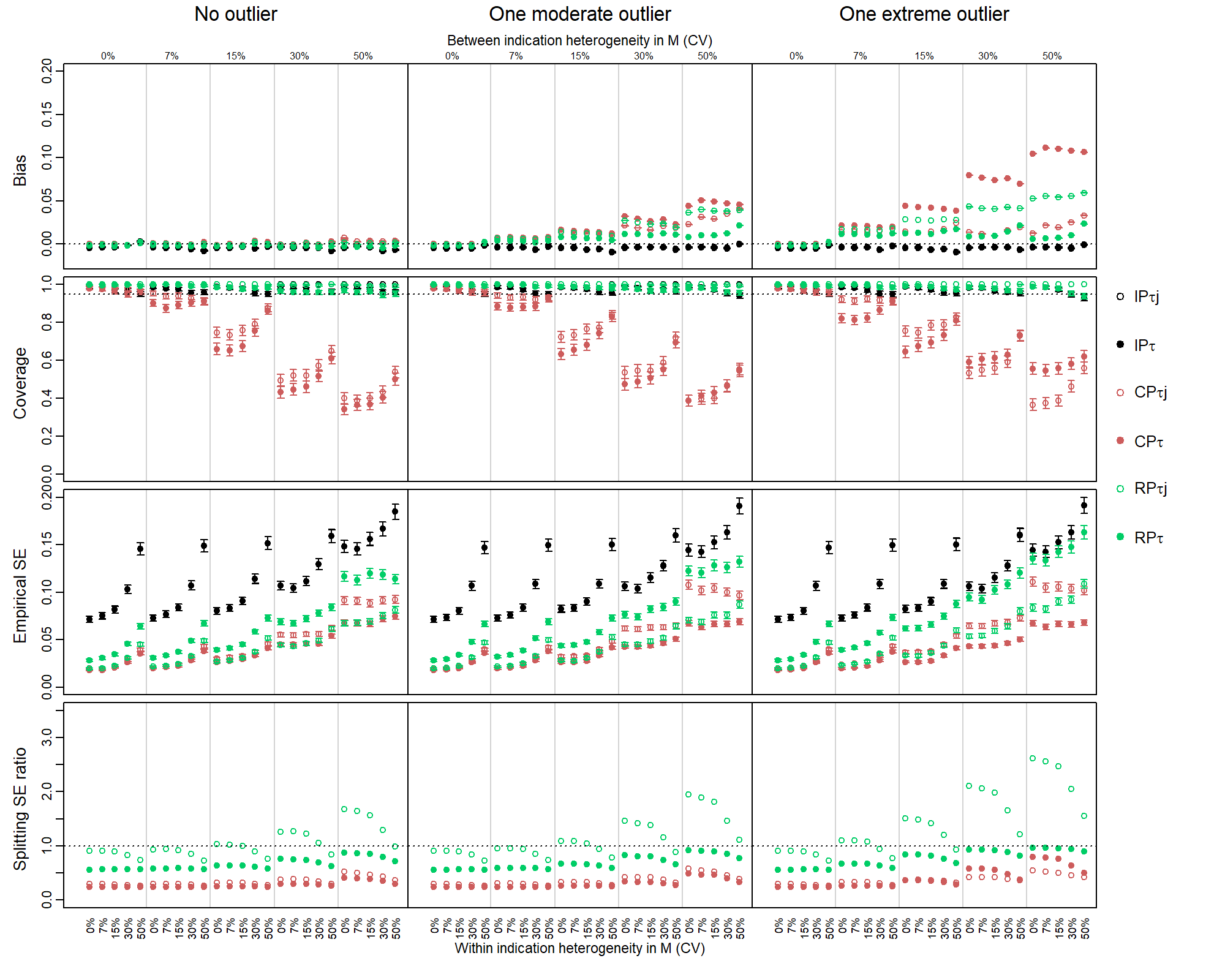


**Small dataset**


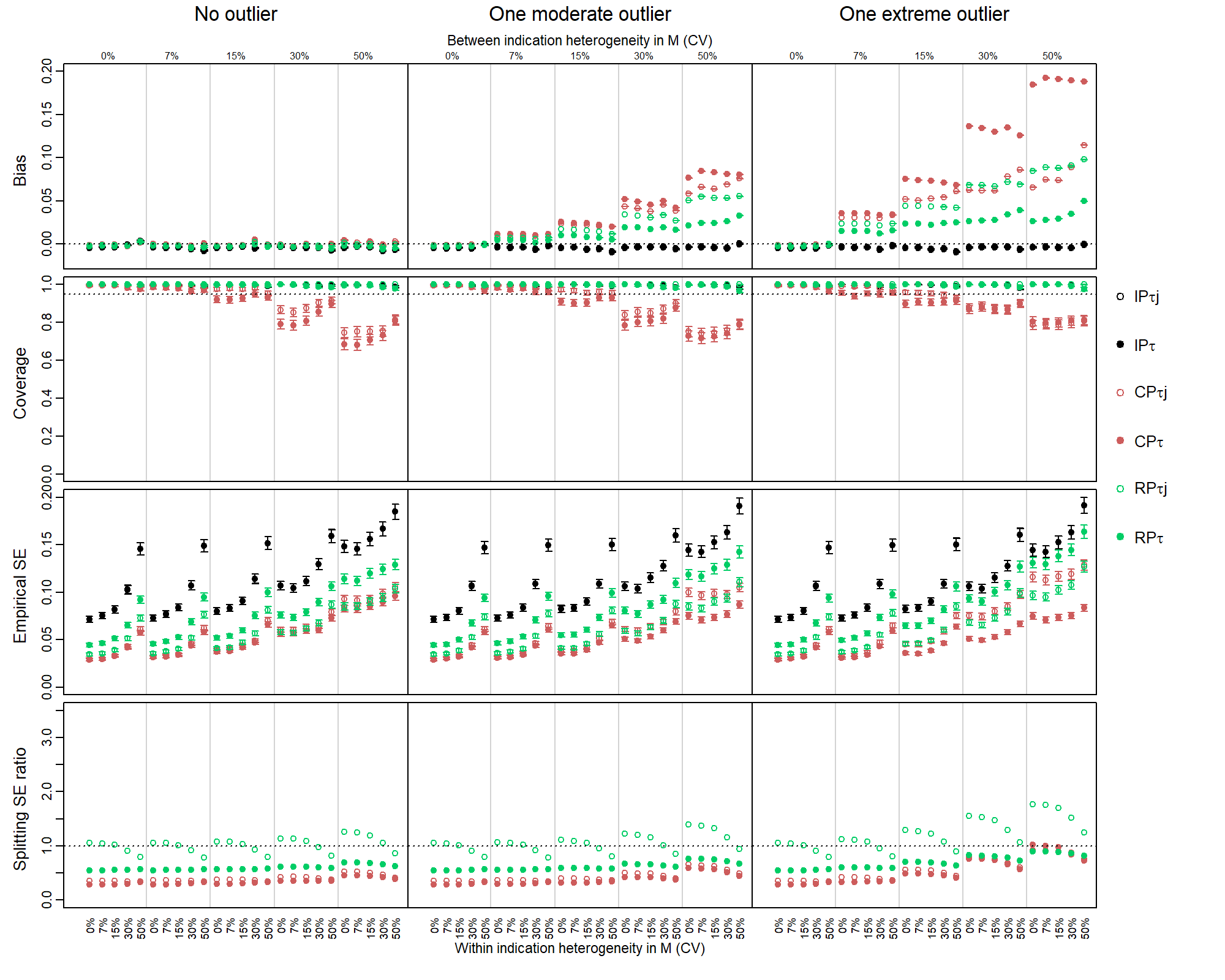


### Univariate mixture

**Large dataset**


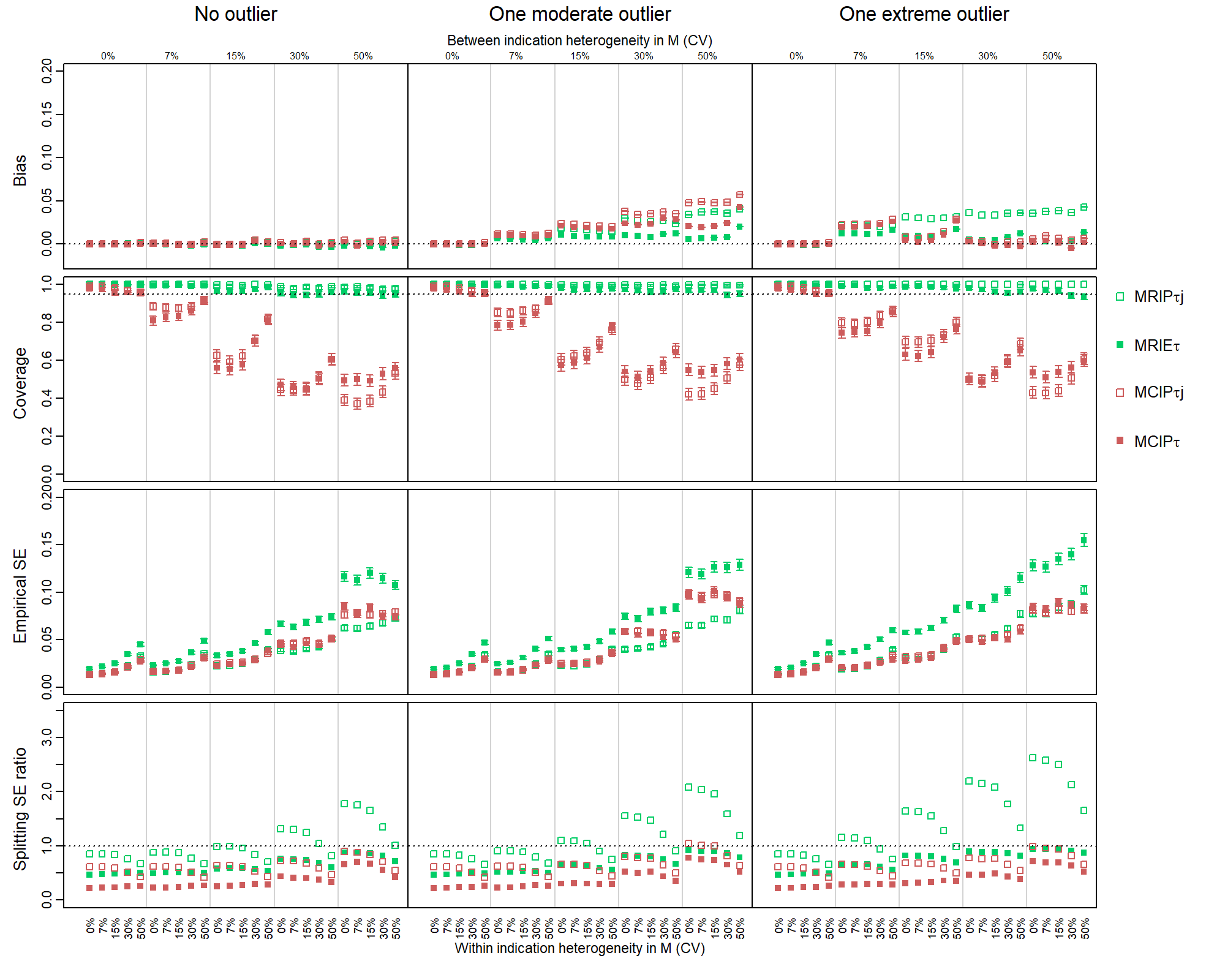


**Medium dataset**


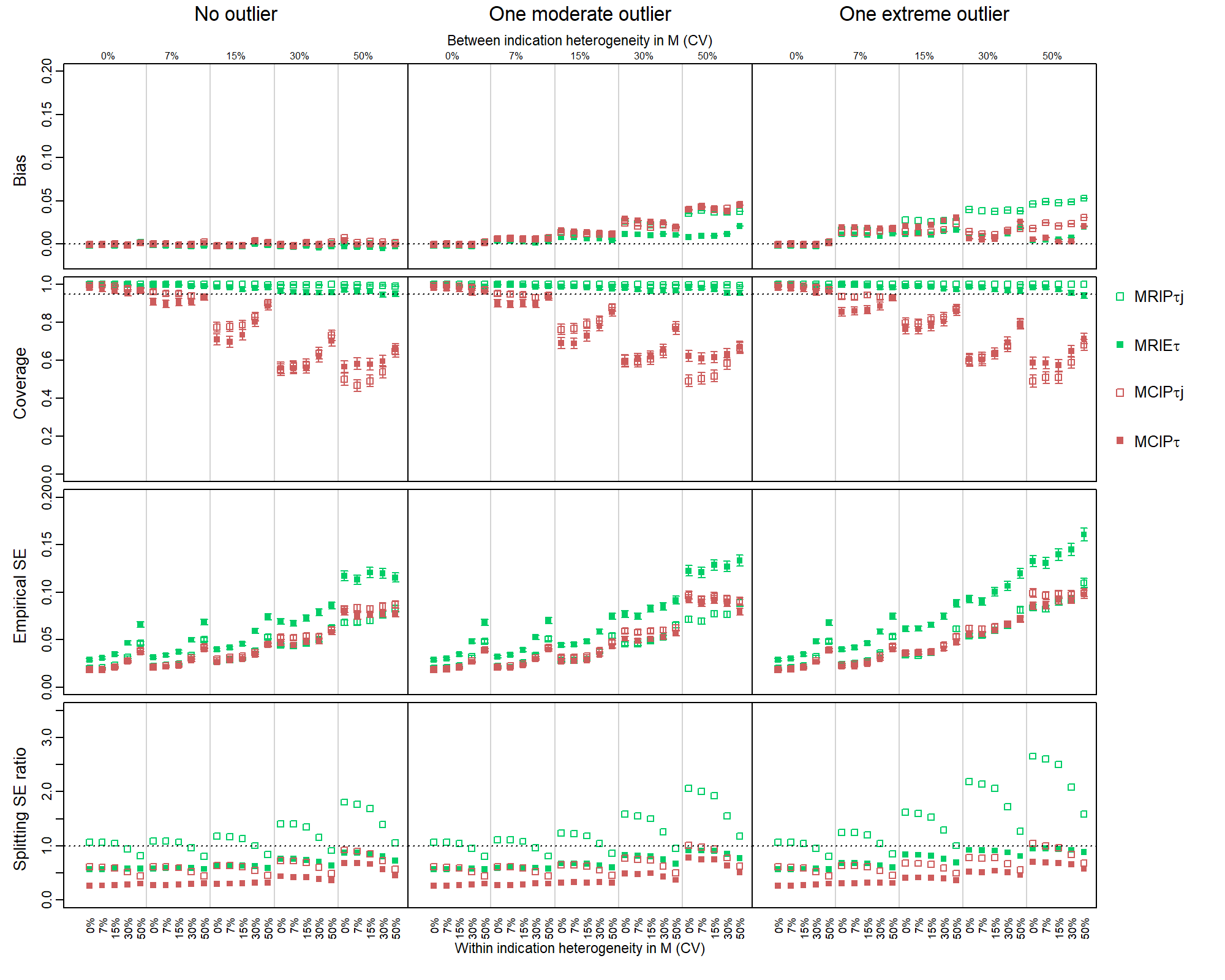


**Small dataset**


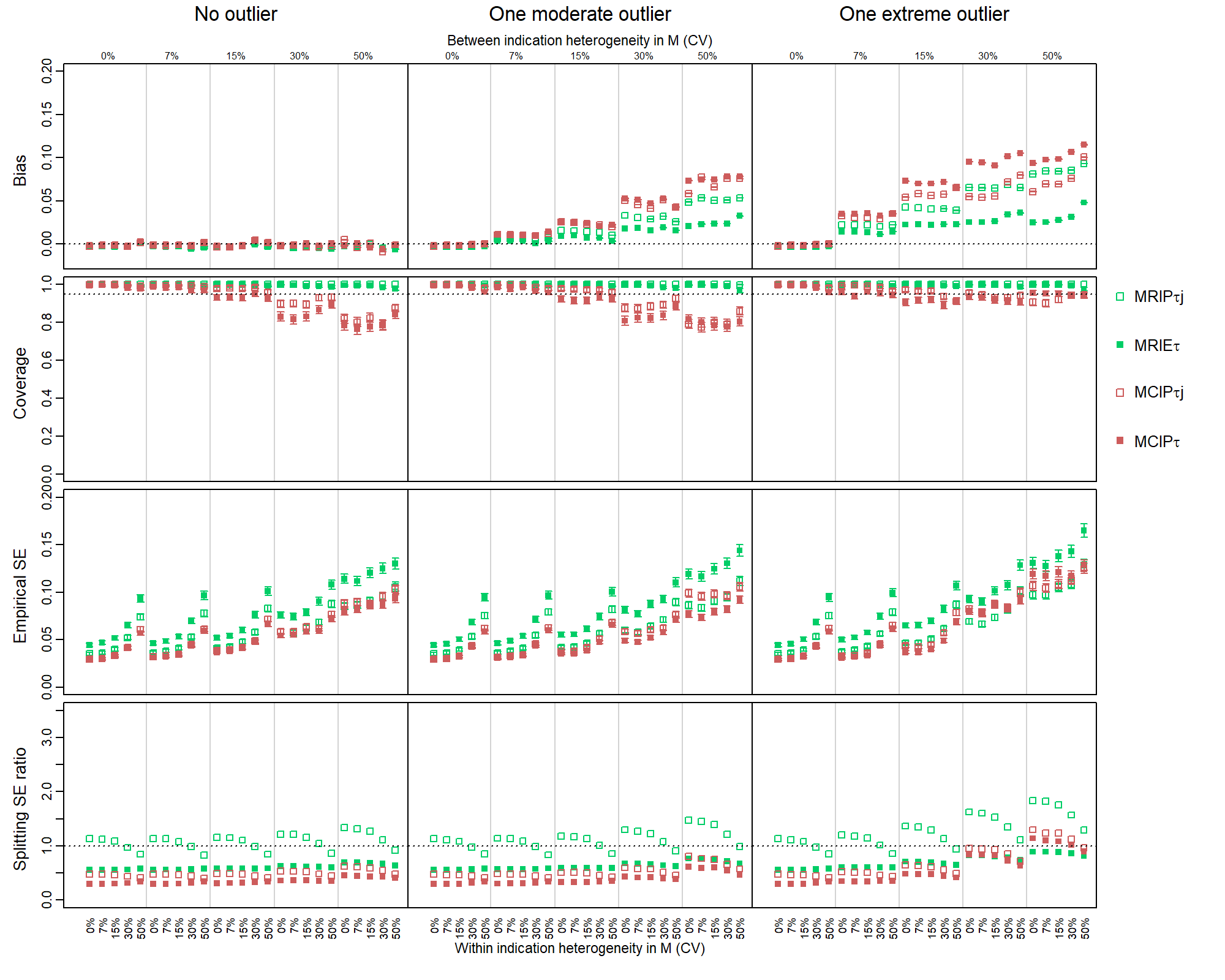


### Surrogate unmatched

**Large dataset**


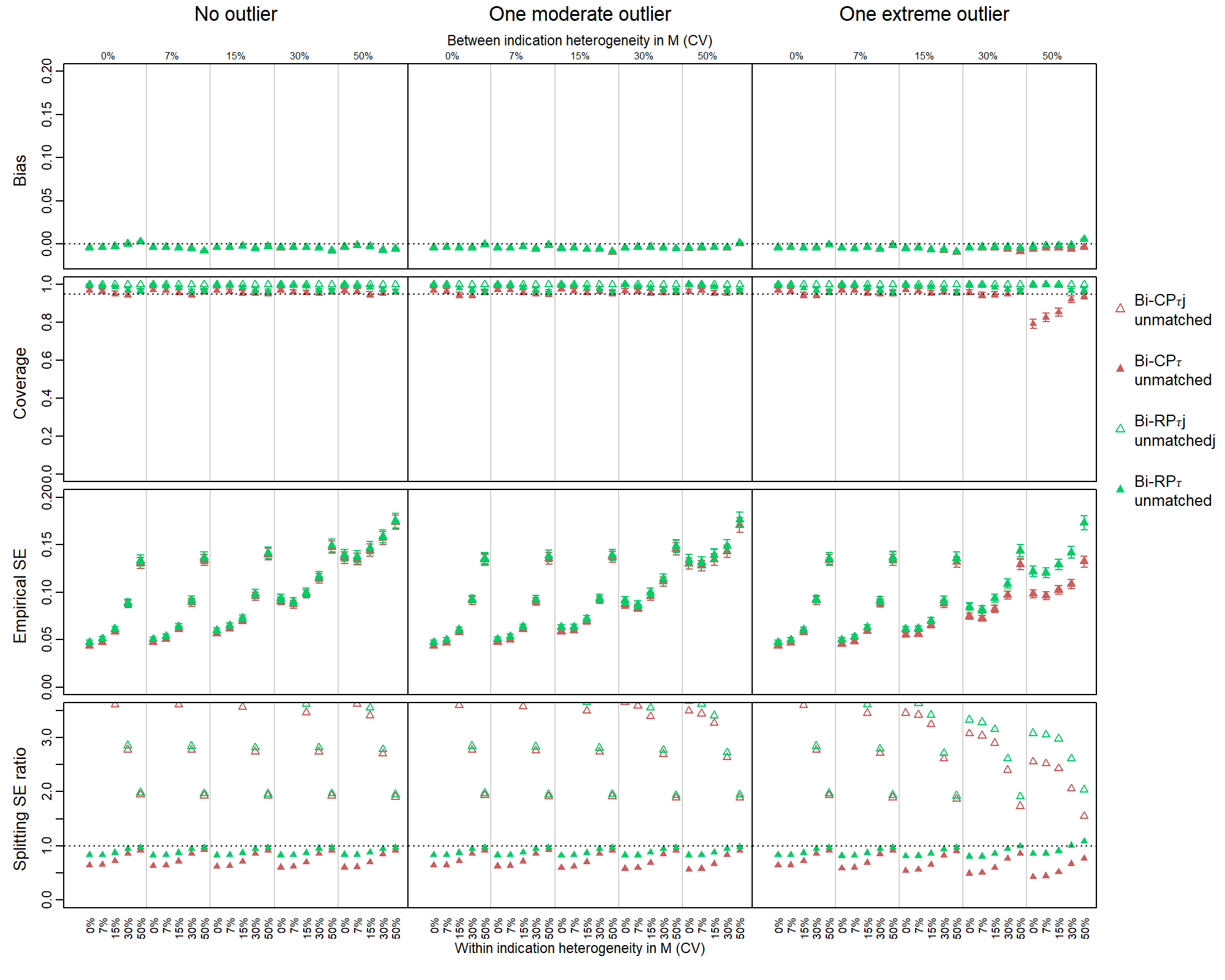


**Medium dataset**


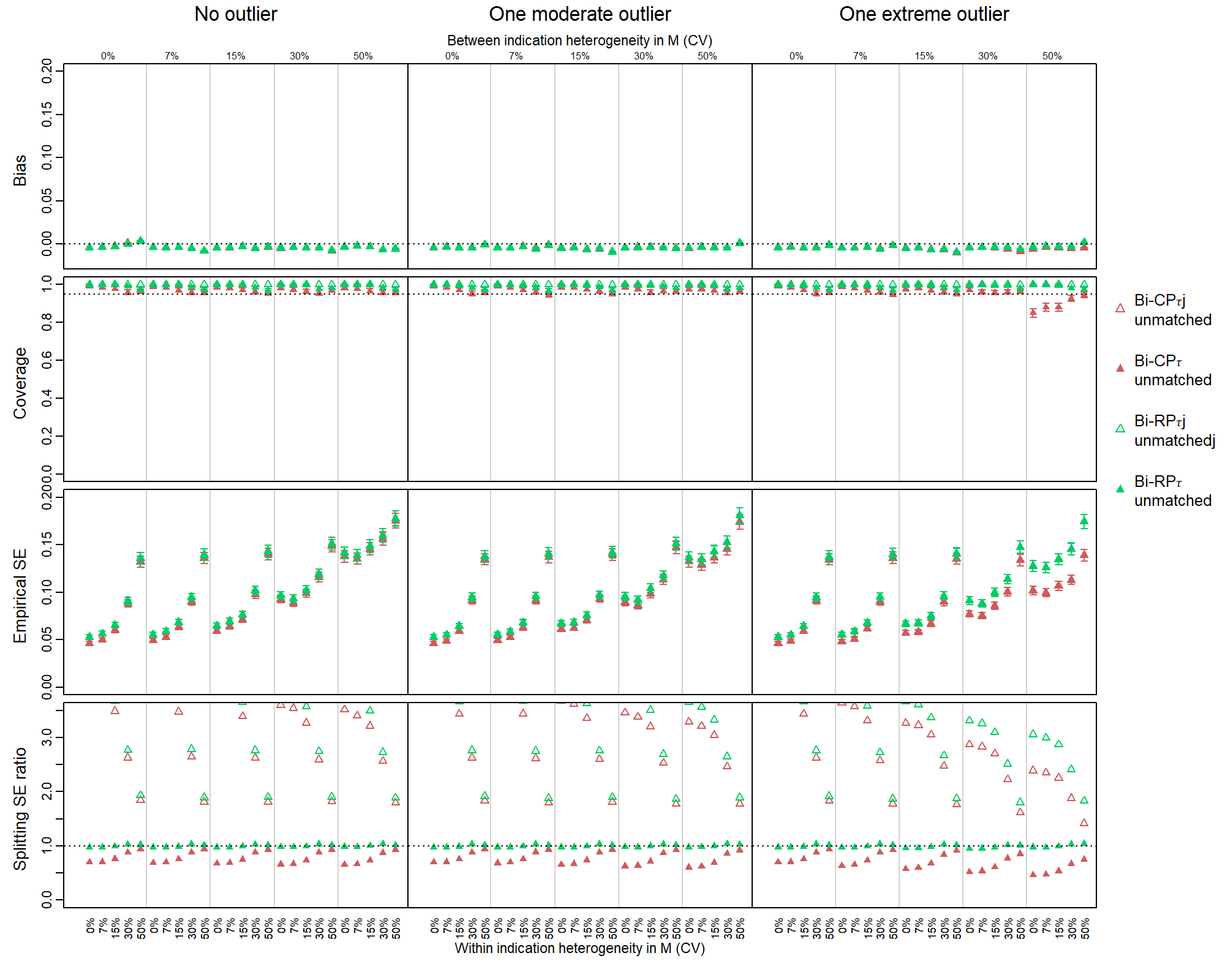


**Small dataset**


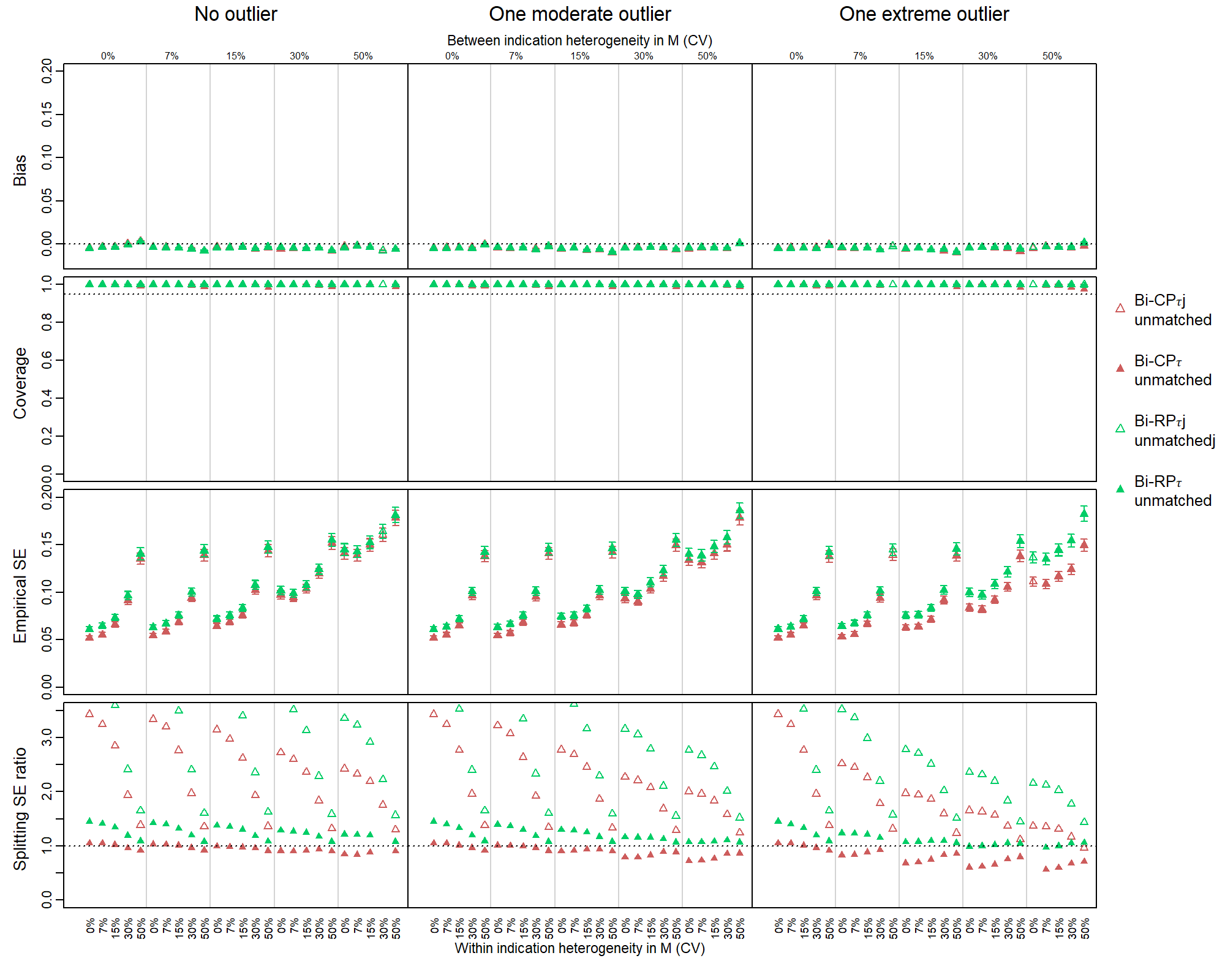


### Surrogate matched

**Large dataset**


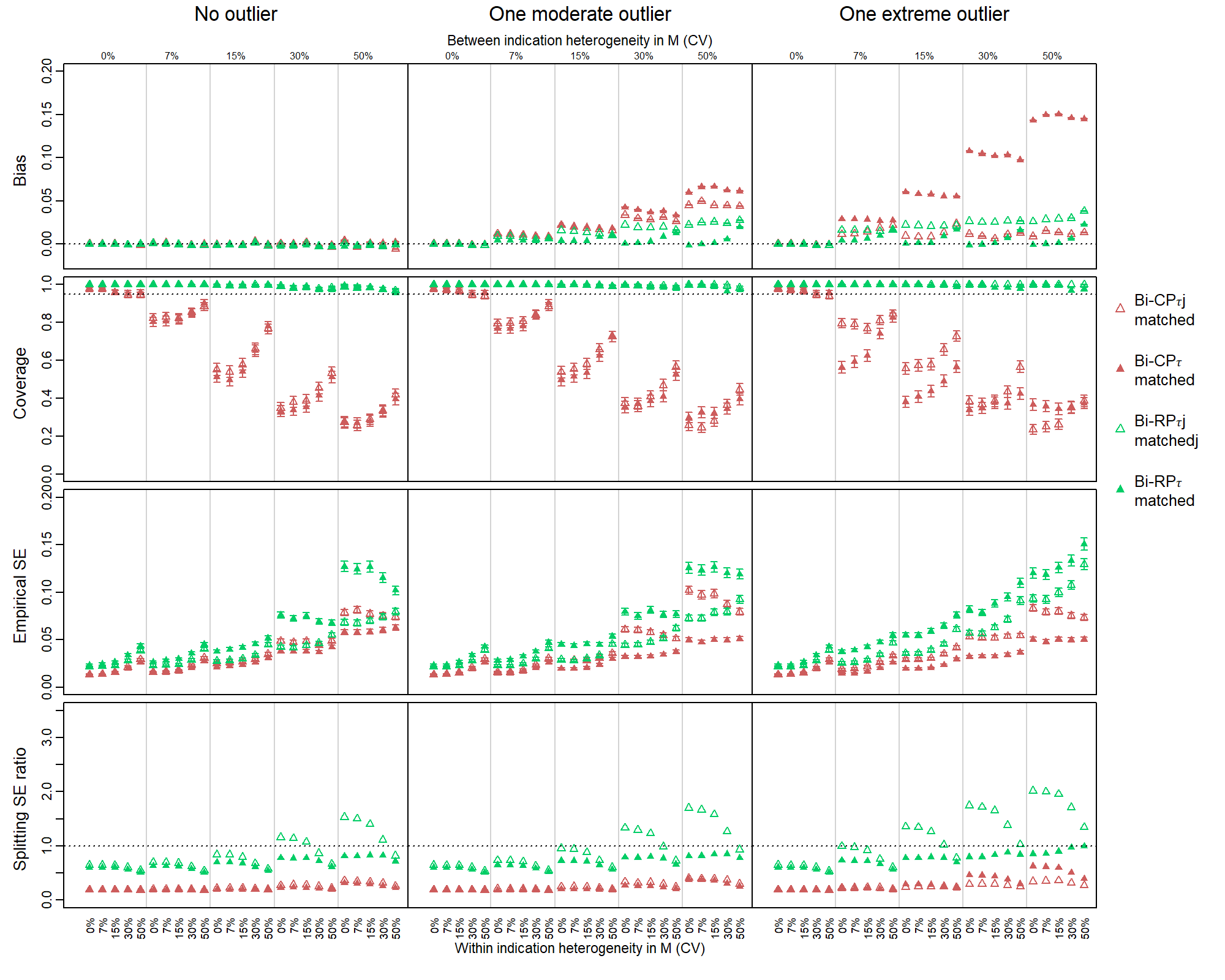


**Medium dataset**


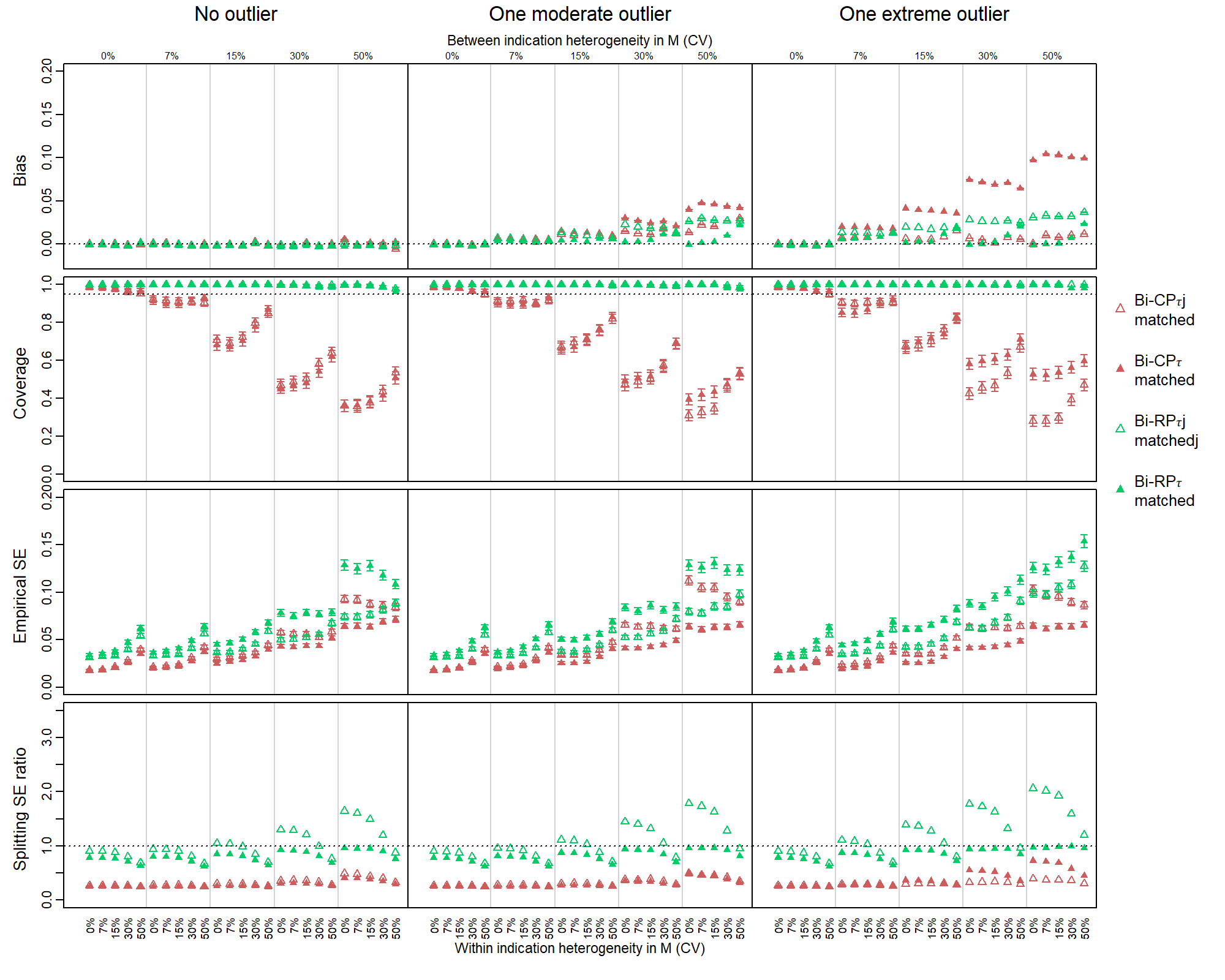


**Small dataset**


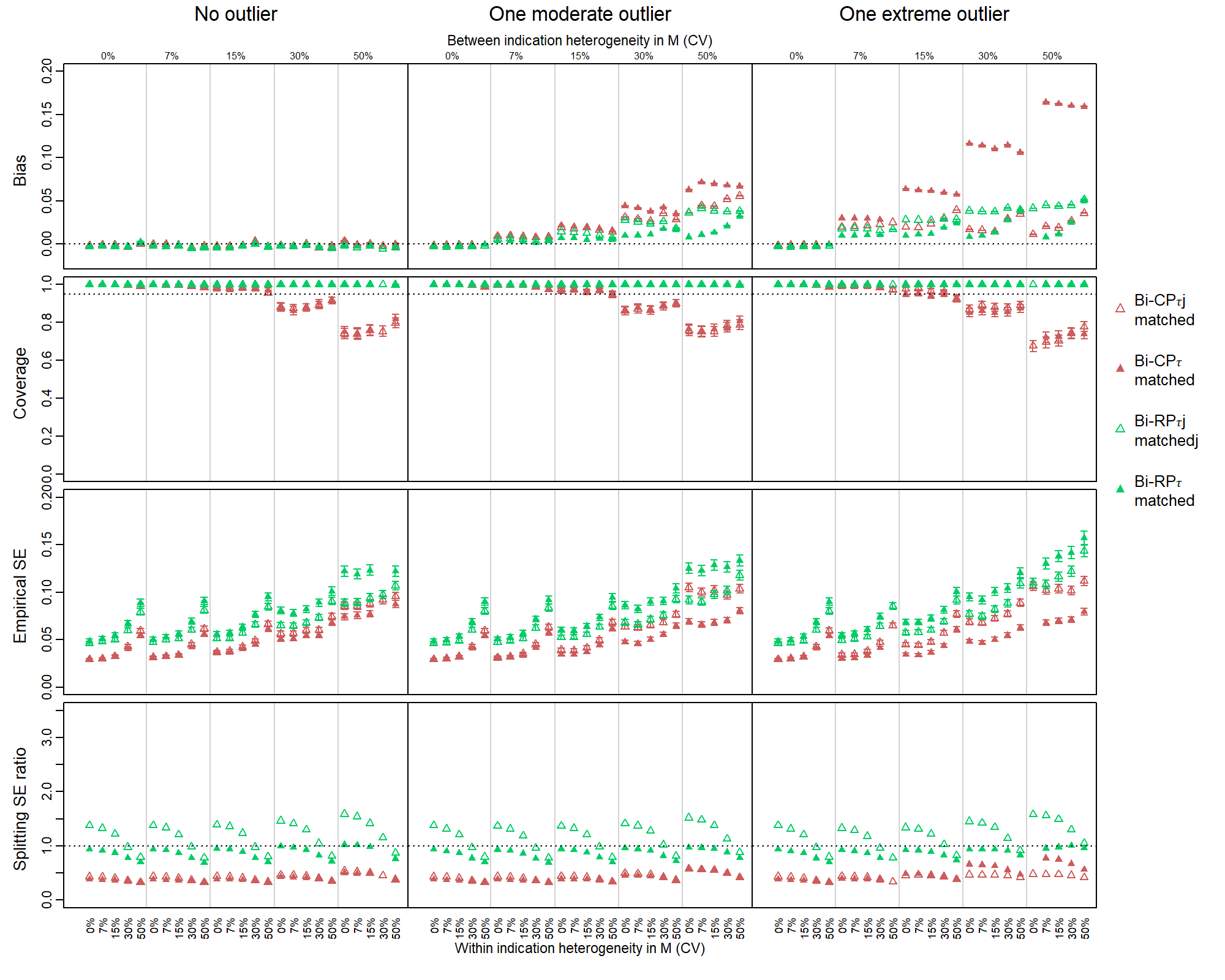


## A4.1.2 No overall survival in target indication

Note that because there is no OS in the target indication it is not possible to esimtate the univariate IP model. This causes issues with the interpretation of the splitting SE ratio because this is based on the IP model in the target indication. In the graphs below the splitting SE ratio is calculated comparing the SE of the sharing models to the SE of the IP model if there was OS in the target indication.

### Univariate non-mixture

**Large dataset**


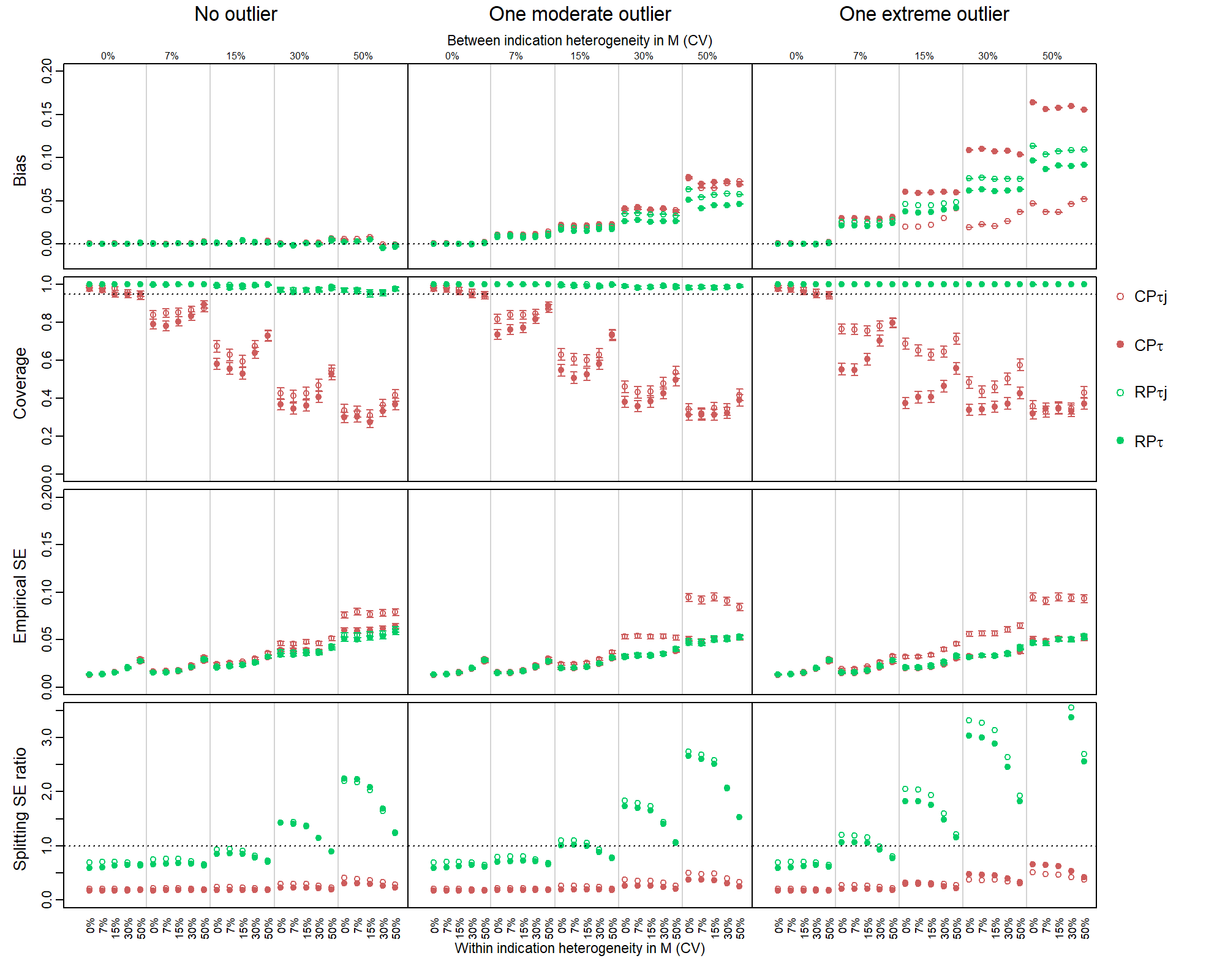


**Medium dataset**


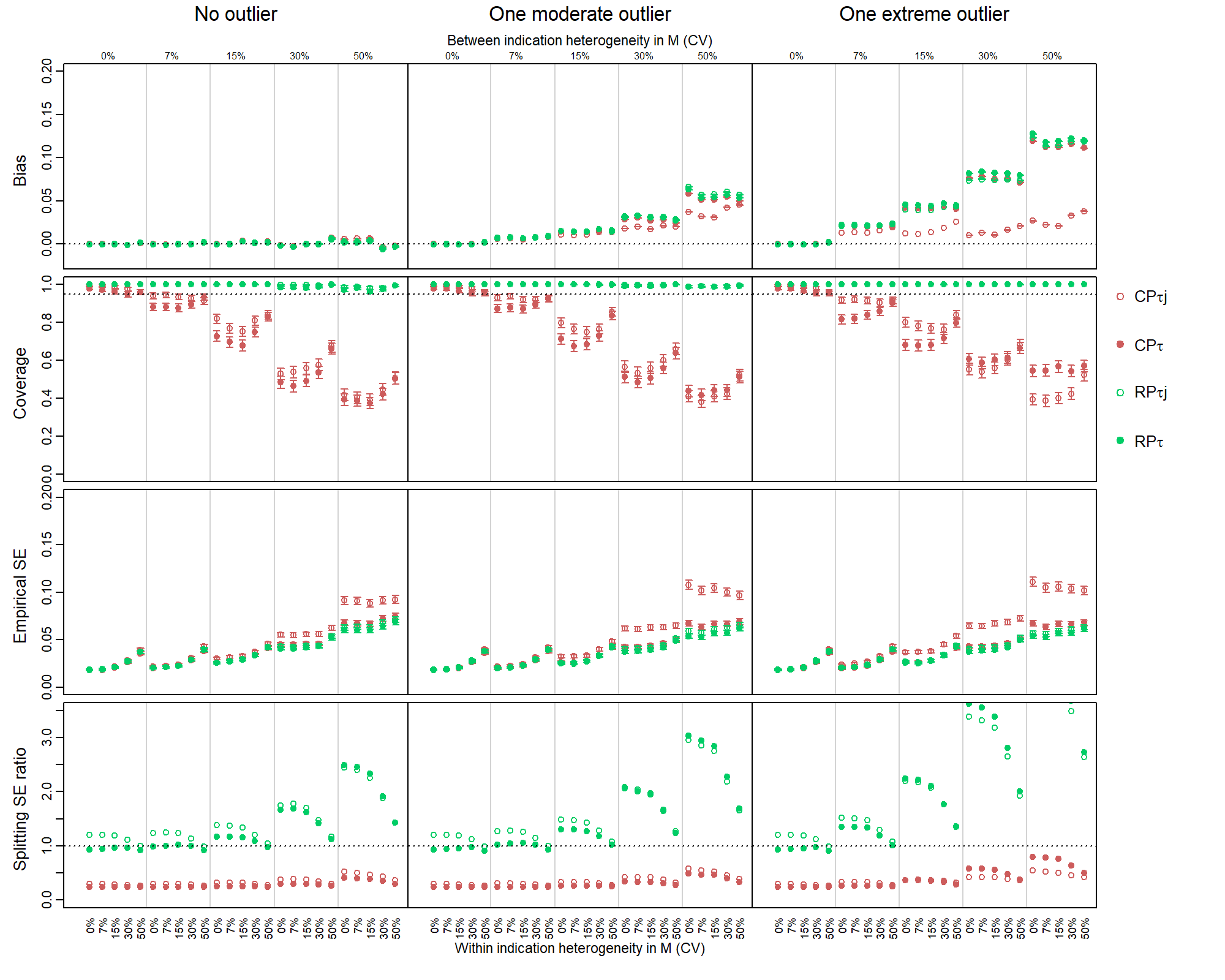


**Small dataset**


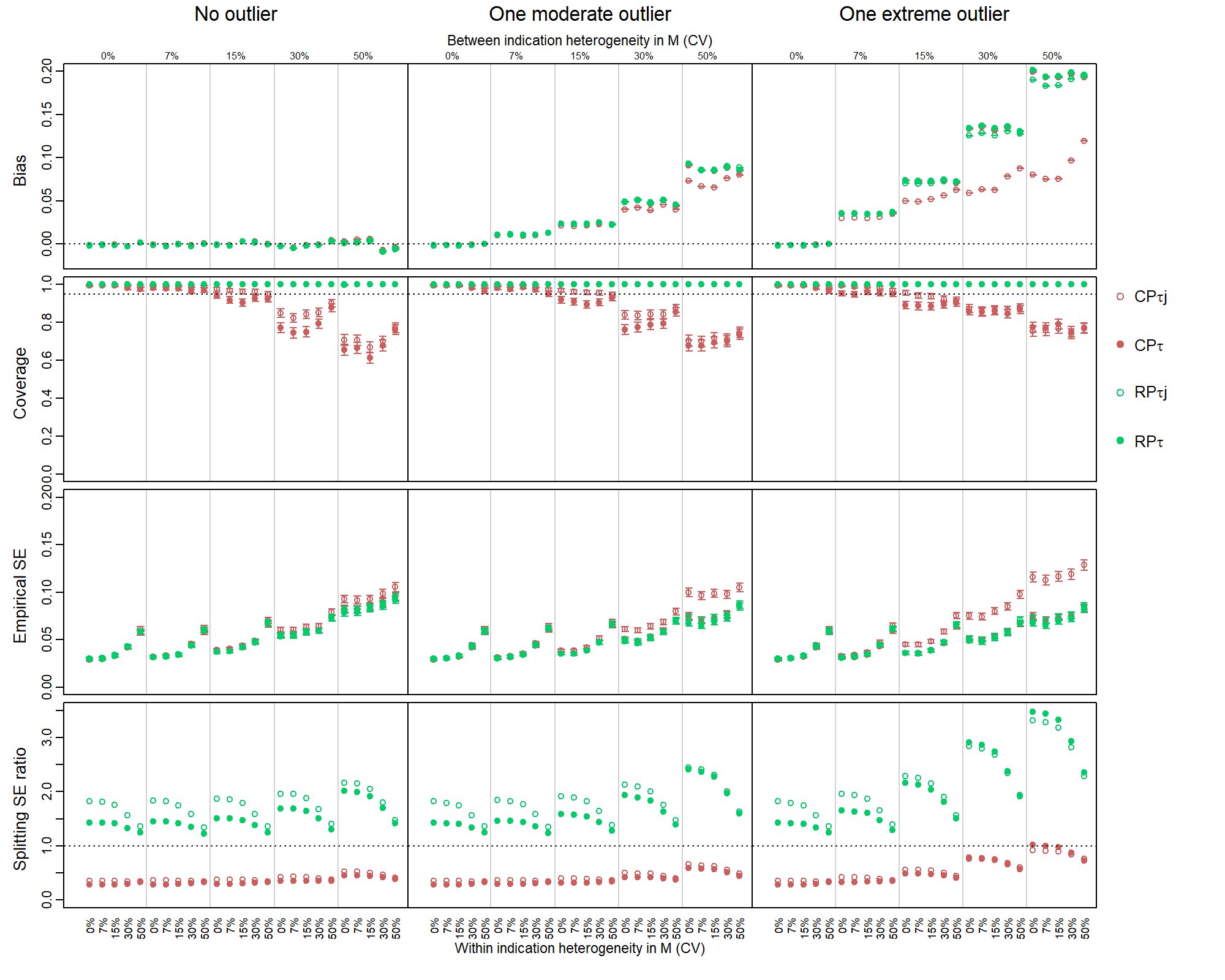


### Univariate mixture

**Large dataset**


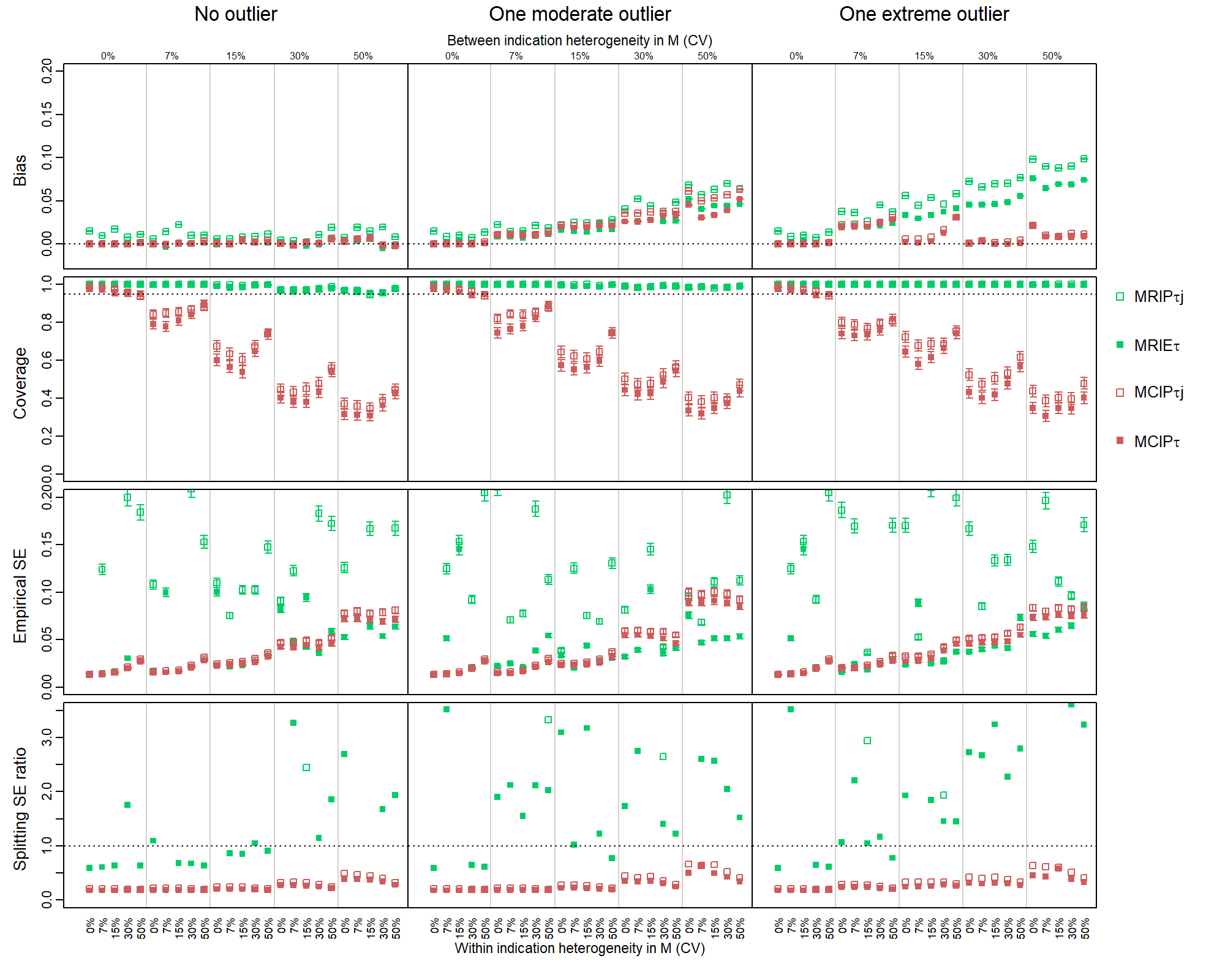


**Medium dataset**


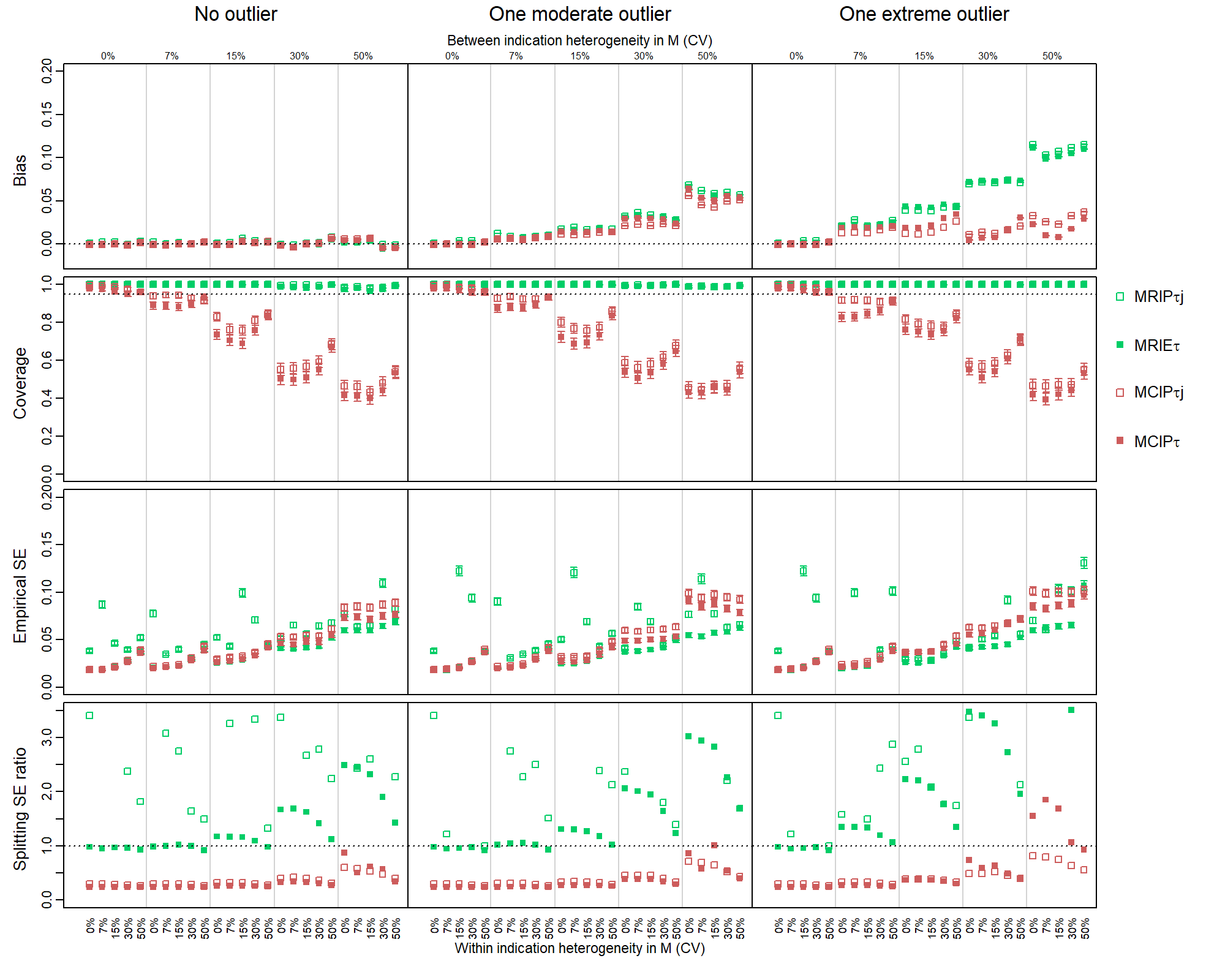


**Small dataset**


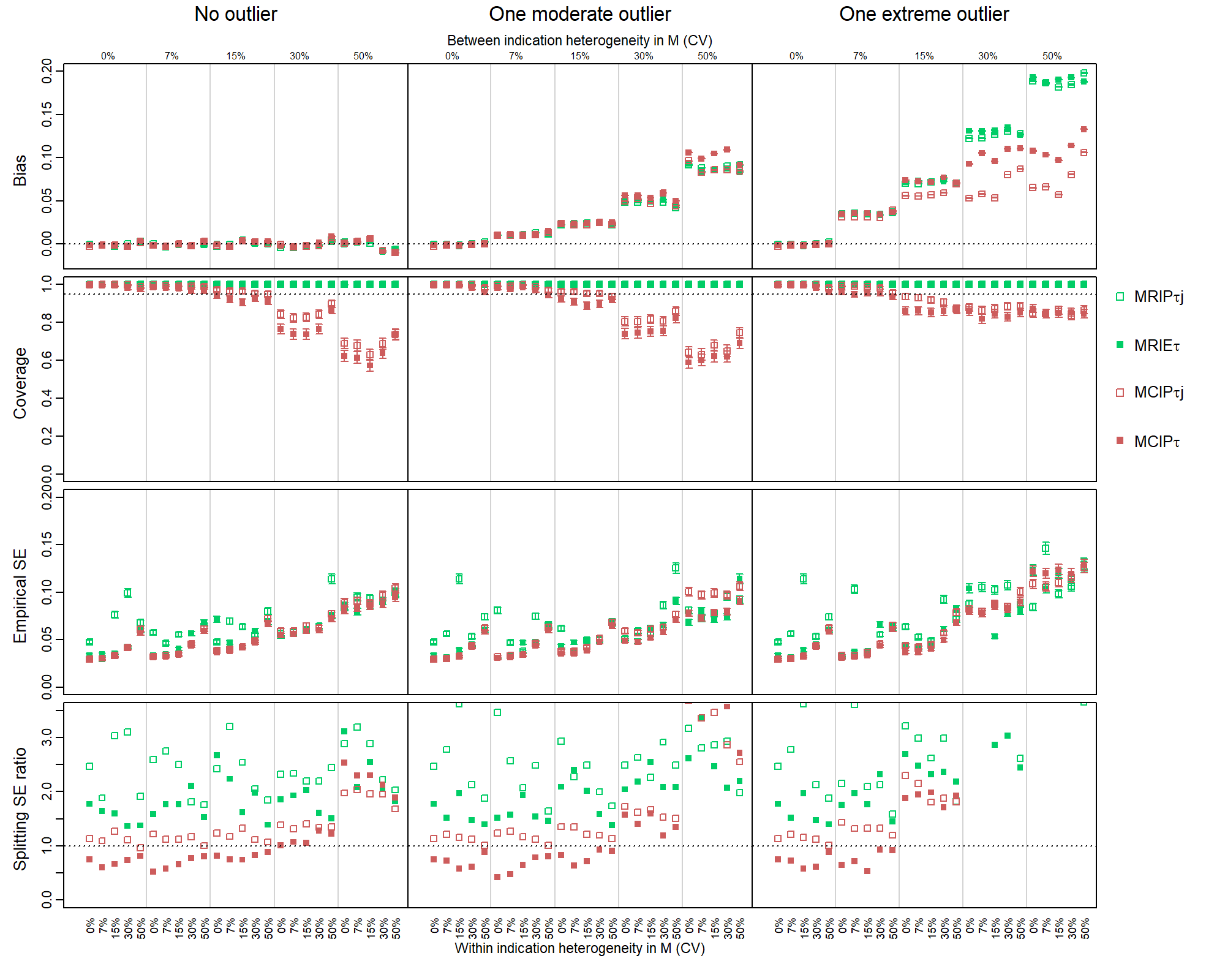


### Surrogate unmatched

**Large dataset**


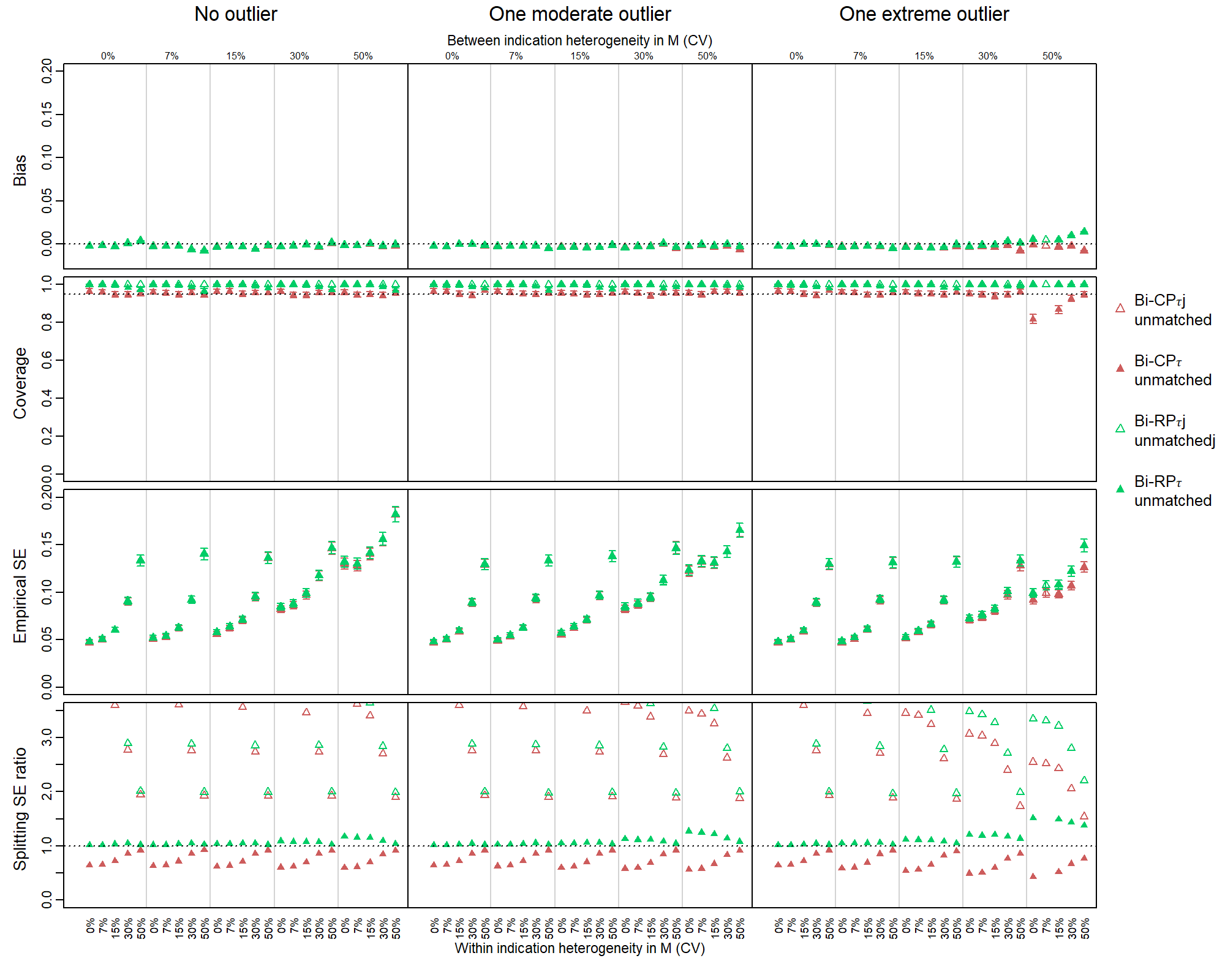


**Medium dataset**


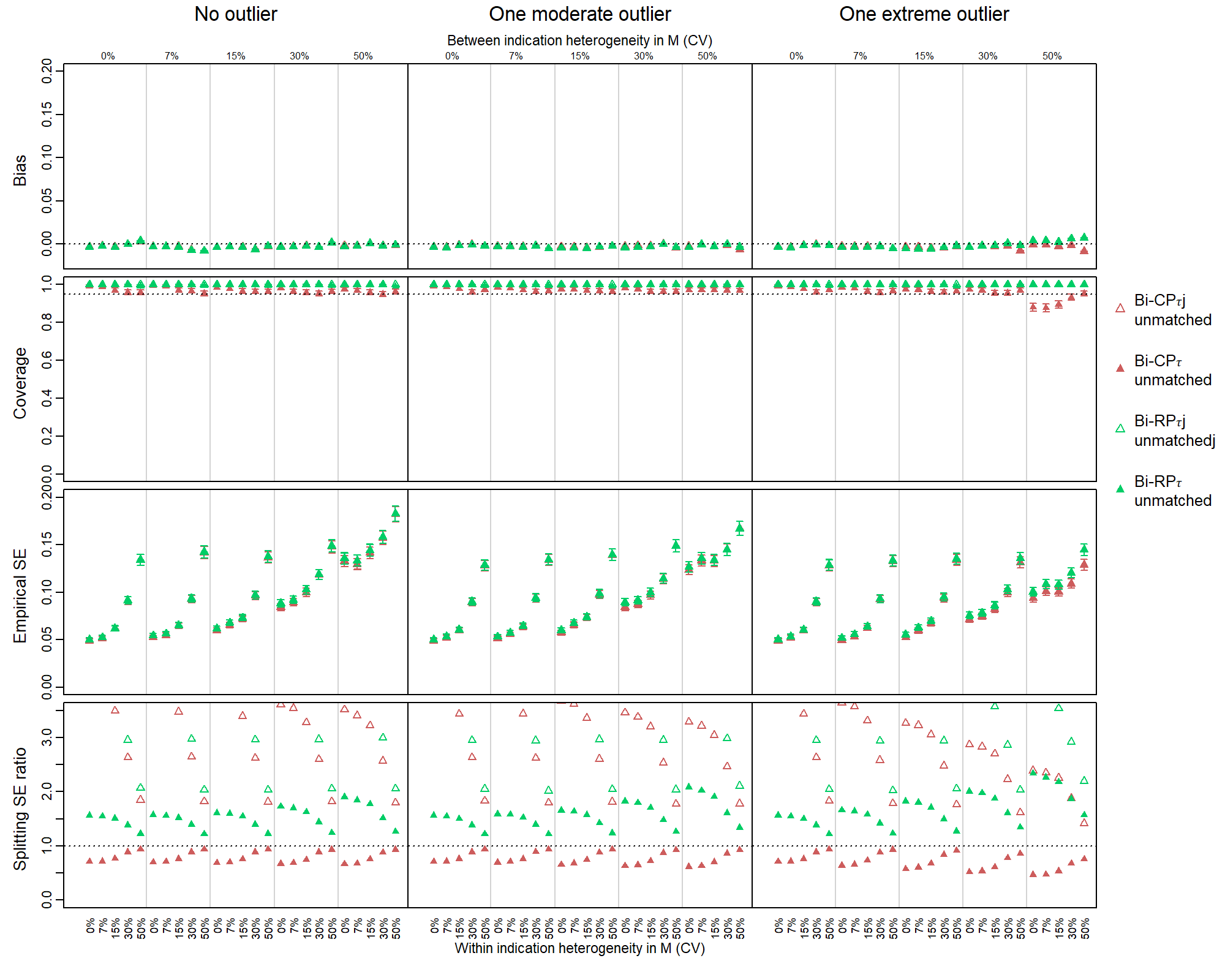


**Small dataset**


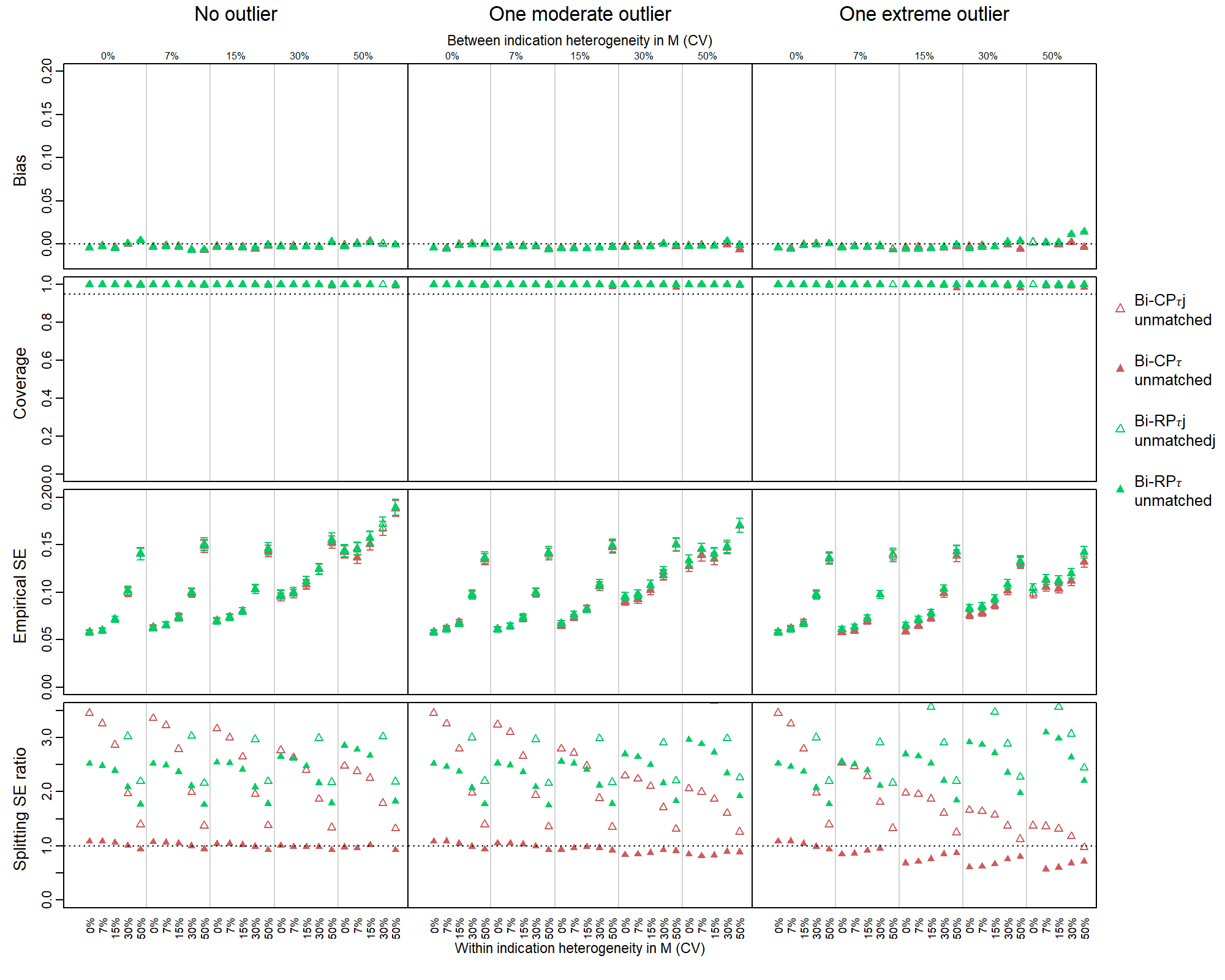


### Surrogate matched

**Large dataset**


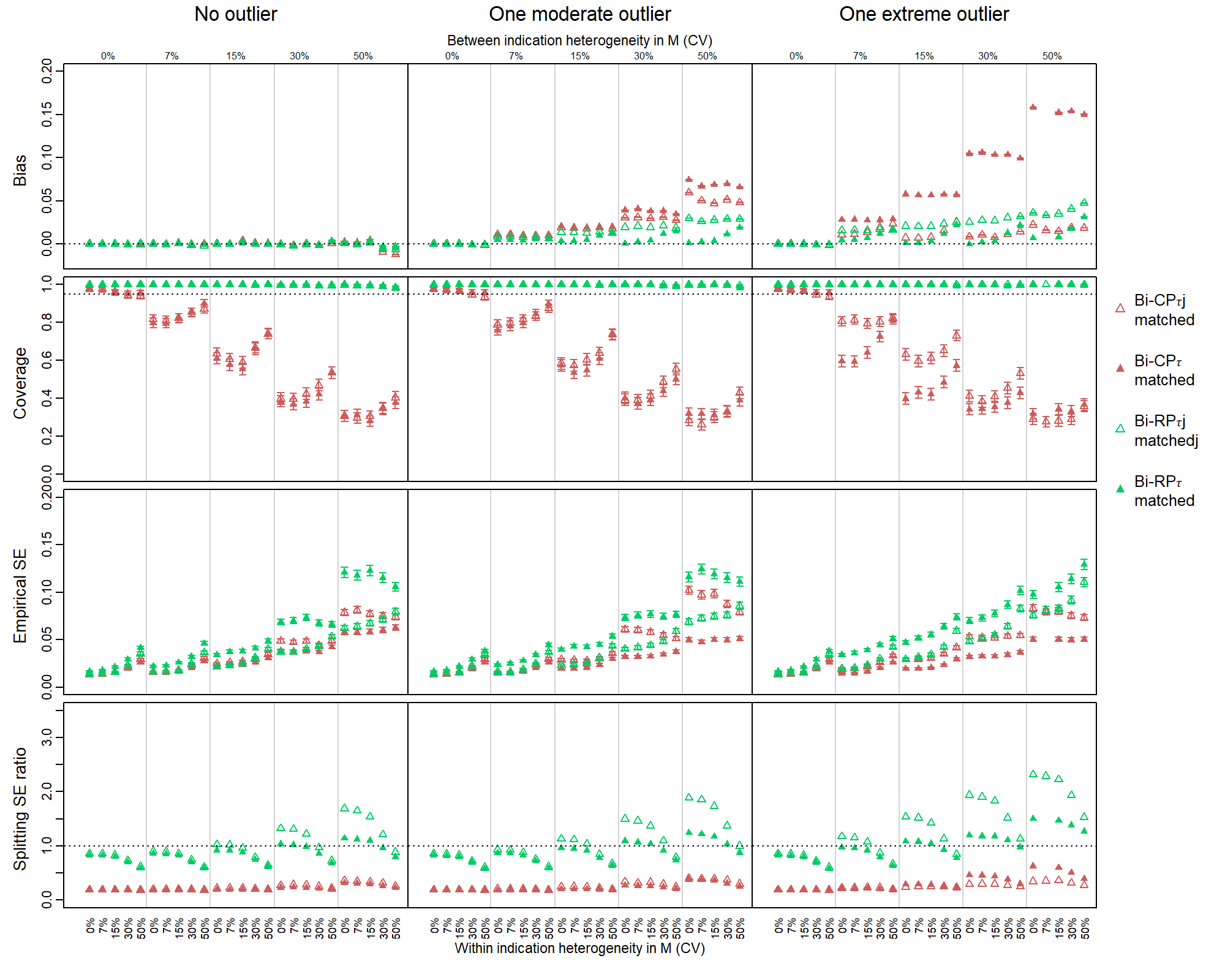


**Medium dataset**


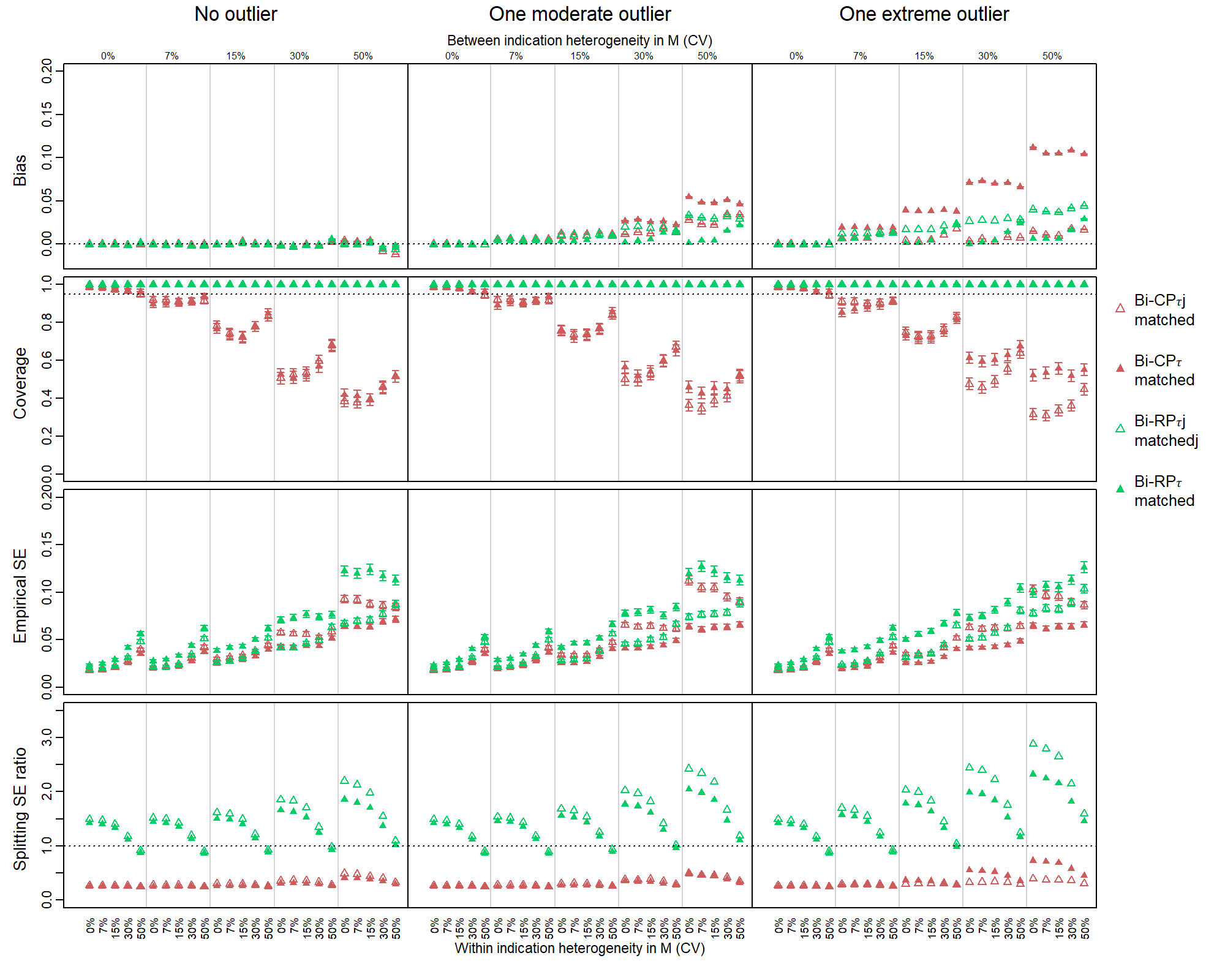


**Small dataset**


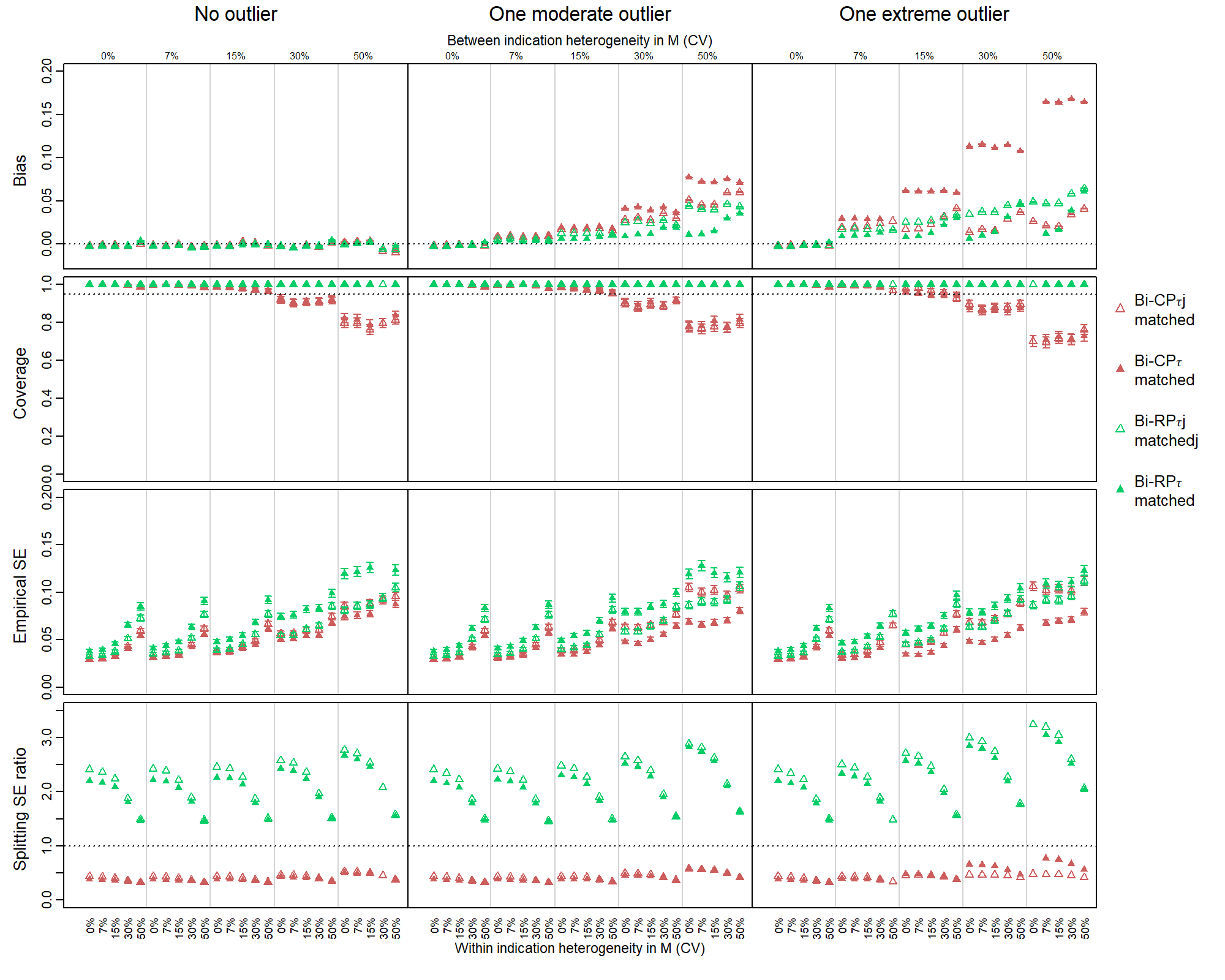


# A4.2 Outlier target indication

Note that the mixture models were not fit to this dataset.

## A4.2.1 With overall survival in target indication

### Univariate non-mixture


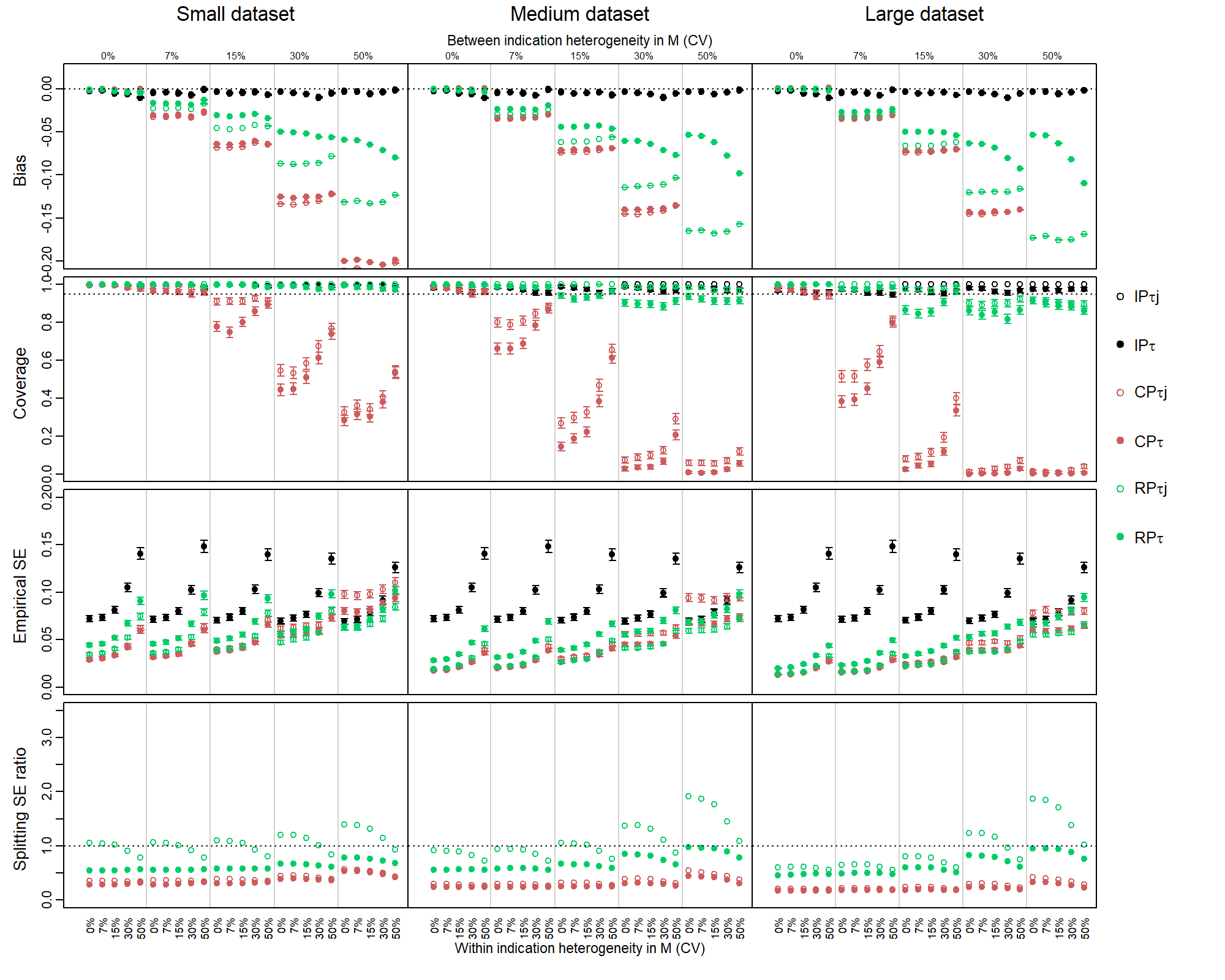


### Surrogate unmatched


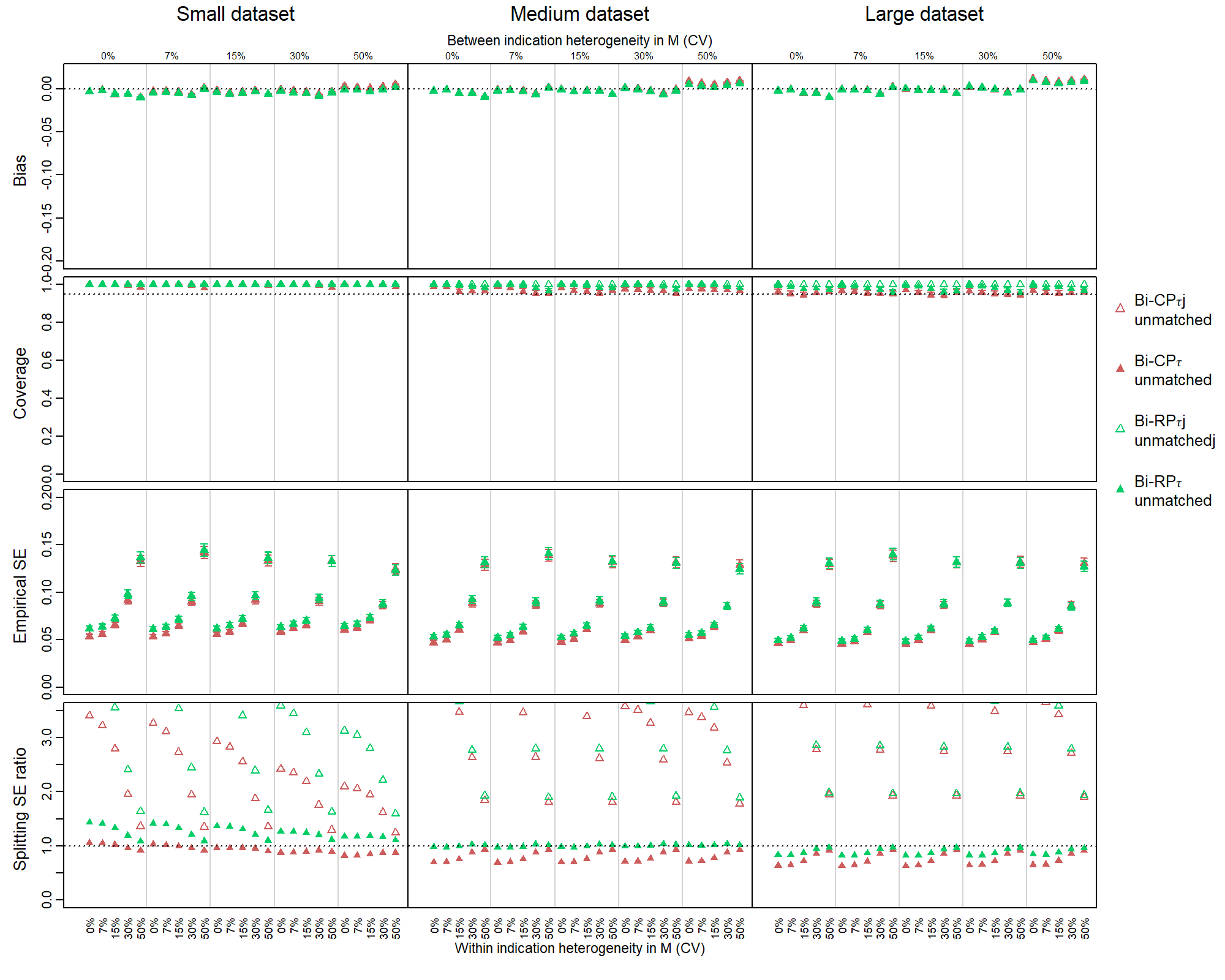


### Surrogate matched


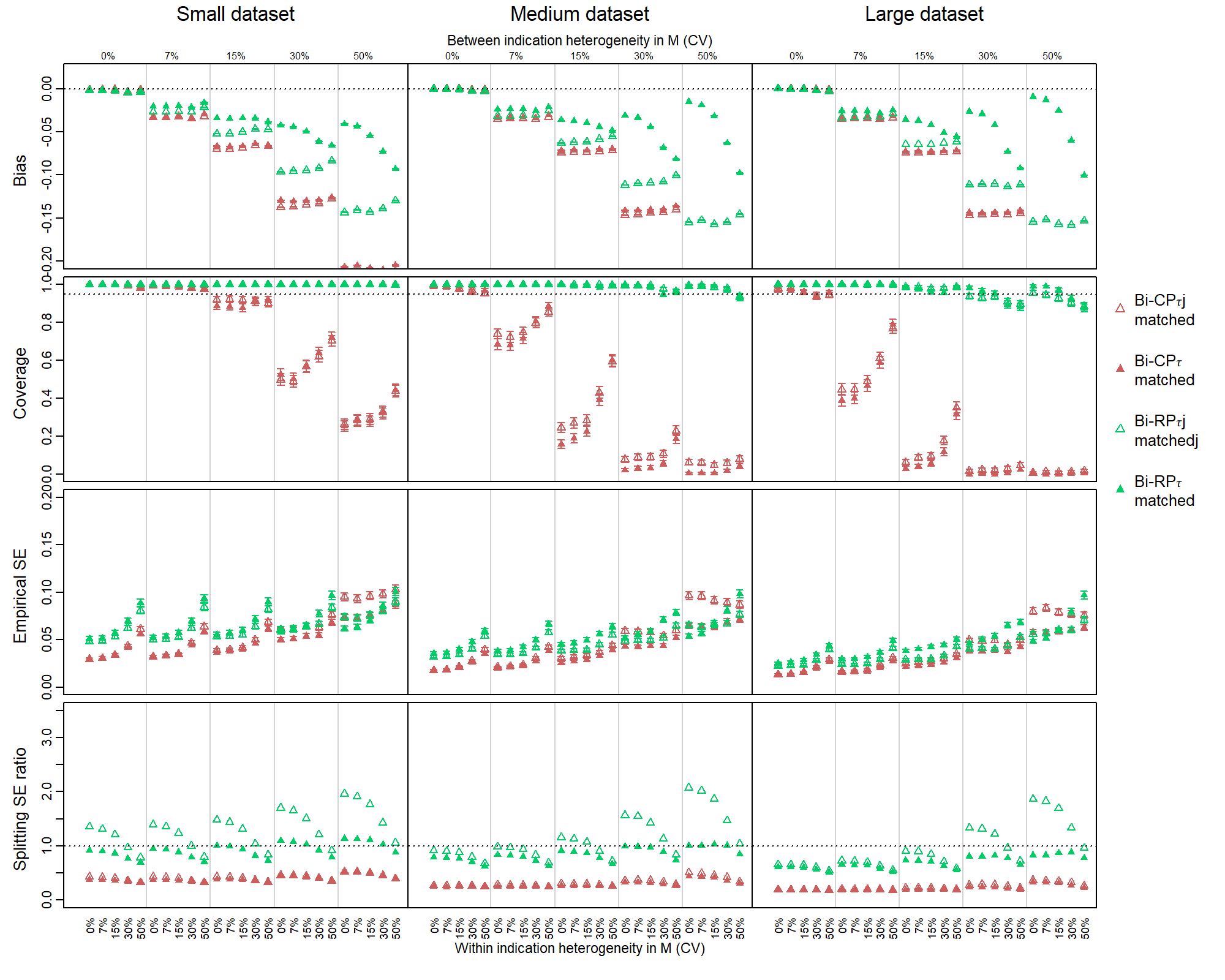


## A4.2.2 No overall survival in target indication

Note that because there is no OS in the target indication it is not possible to estimate the univariate IP model. This causes issues with the interpretation of the splitting SE ratio because this is based on the IP model in the target indication. In the graphs below the splitting SE ratio is calculated comparing the SE of the sharing models to the SE of the IP model if there was OS in the target indication.

### Univariate non-mixture


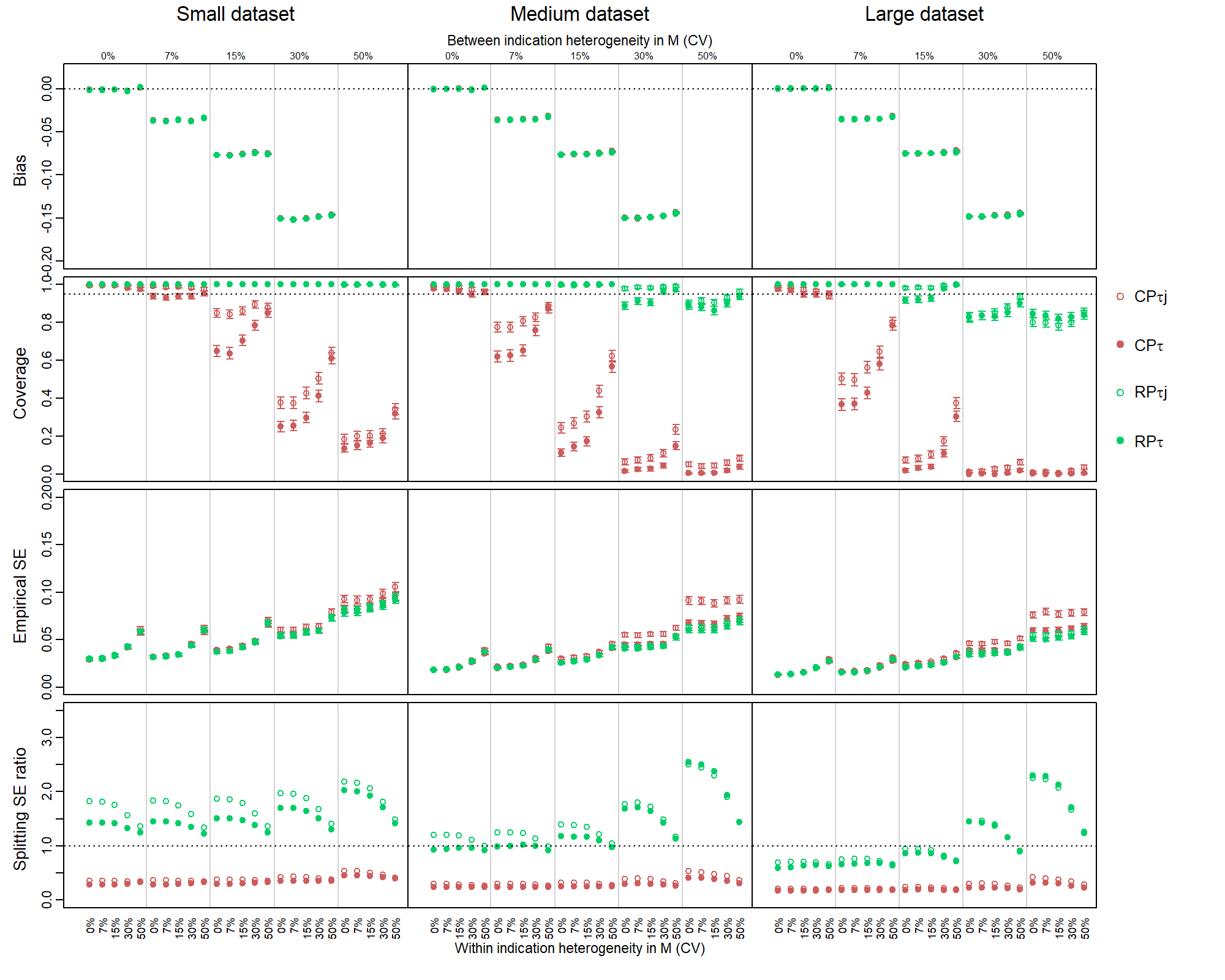


### Surrogate unmatched


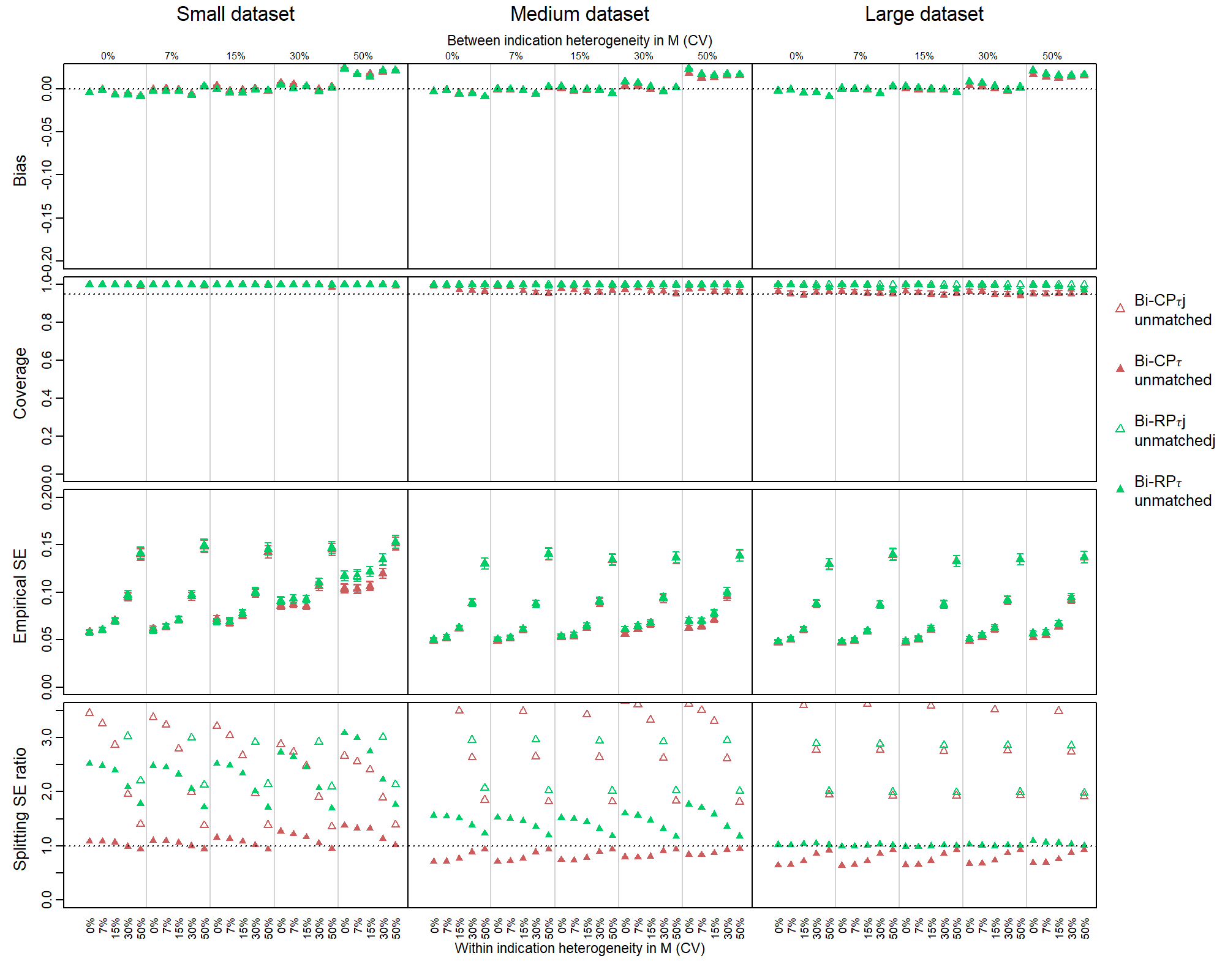


### Surrogate matched


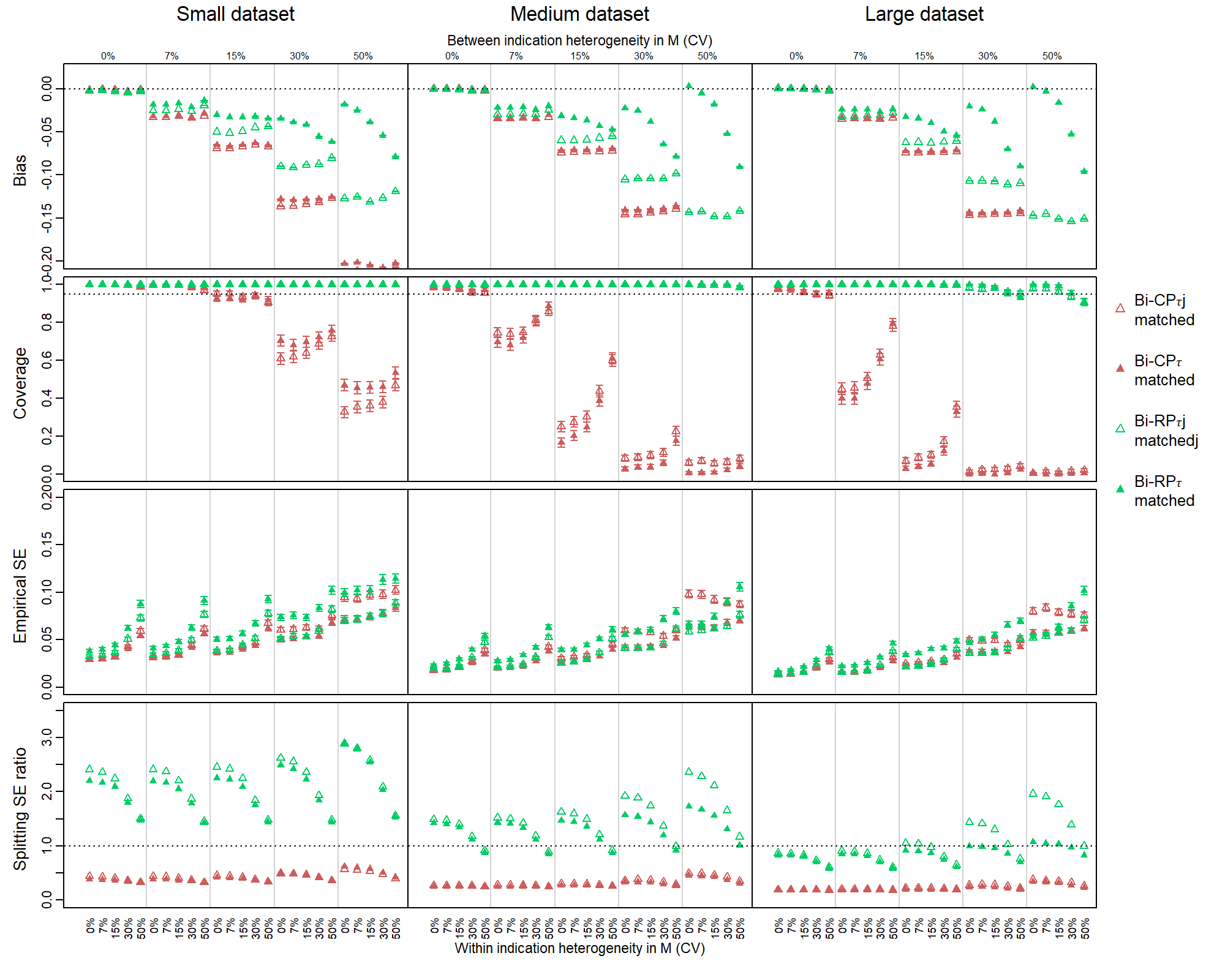


# References

Erdmann, A., J. Beyersmann and K. Rufibach (2025). "Oncology Clinical Trial Design Planning Based on a Multistate Model That Jointly Models Progression‐Free and Overall Survival Endpoints." Biometrical Journal **67**(1): e70017.

Jansen, J. P., D. Incerti and T. A. Trikalinos (2023). "Multi‐state network meta‐analysis of progression and survival data." Statistics in medicine **42**(19): 3371-3391.

Singh, J., S. Anwer, S. Palmer, P. Saramago, A. Thomas, S. Dias, M. Soares and S. Bujkiewicz (2023). "Multi-indication evidence synthesis in oncology health technology assessment." arXiv preprint arXiv:2311.12452.
